# A novel *Plasmodium falciparum* rhoptry associated adhesin mediates erythrocyte invasion through the sialic-acid dependent pathway

**DOI:** 10.1038/srep29185

**Published:** 2016-07-07

**Authors:** Gaurav Anand, K. Sony Reddy, Alok Kumar Pandey, Syed Yusuf Mian, Hina Singh, Shivani Arora Mittal, Emmanuel Amlabu, Quique Bassat, Alfredo Mayor, Virander Singh Chauhan, Deepak Gaur

**Affiliations:** 1 Malaria Group, International Centre for Genetic Engineering and Biotechnology (ICGEB), New Delhi, India; 2 Laboratory of Malaria and Vaccine Research, School of Biotechnology, Jawaharlal Nehru University, New Delhi, India; 3 ISGlobal, Barcelona Ctr. Int. Health Res. (CRESIB), Hospital Clínic - Universitat de Barcelona, Barcelona, Spain; 4 Centro de Investigação em Saude de Manhiça (CISM), Maputo, Mozambique

## Abstract

Erythrocyte invasion by *Plasmodium falciparum* merozoites is central to blood-stage infection and malaria pathogenesis. This intricate process is coordinated by multiple parasite adhesins that bind erythrocyte receptors and mediate invasion through several alternate pathways. *P. falciparum* expresses 2700 genes during the blood-stages, of which the identity and function of many remains unknown. Here, we have identified and characterized a novel *P. falciparum* rhoptry associated adhesin (PfRA) that mediates erythrocyte invasion through the sialic-acid dependent pathway. PfRA appears to play a significant functional role as it is conserved across different *Plasmodium* species. It is localized in the rhoptries and further translocated to the merozoite surface. Both native and recombinant PfRA specifically bound erythrocytes in a sialic-acid dependent, chymotrypsin and trypsin resistant manner, which was abrogated by PfRA antibodies confirming a role in erythrocyte invasion. PfRA antibodies inhibited erythrocyte invasion and in combination with antibodies against other parasite ligands produced an additive inhibitory effect, thus validating its important role in erythrocyte invasion. We have thus identified a novel *P. falciparum* adhesin that binds with a sialic acid containing erythrocyte receptor. Our observations substantiate the strategy to block *P. falciparum* erythrocyte invasion by simultaneously targeting multiple conserved merozoite antigens involved in alternate invasion pathways.

Malaria is one of the leading pandemic diseases among developing and underdeveloped countries that accounts for around 214 million disease cases and approximately 438000 deaths worldwide (2015 World Malaria Report). The causative agents of malaria are the protozoan parasites that belong to the genus *Plasmodium*, of which *Plasmodium falciparum* causes the most severe form of malaria, cerebral malaria and is responsible for most global disease mortality^1^. Though current strategies of prevention and treatment have substantially reduced disease burden, the increasing *P. falciparum* resistance to anti-malarial drugs^2^,^3^ and the emergence of insecticide resistant mosquitoes^4^ still poses a major threat. Thus, an effective vaccine would be an effective tool for the control, elimination, or even possible eradication of malaria^5^.

*P. falciparum* completes its complex life cycle through two alternate hosts, the female *Anopheles* mosquito vector and the vertebrate human host^6^. In humans, the *Anopheles* mosquito injects invasive stages, sporozoites that migrate to the liver and infect hepatocytes, where they undergo massive multiplication to form numerous invasive forms, merozoites, which are released into the blood stream. Blood-stage infections are established when *Plasmodium* merozoites invade erythrocytes^7^,^8^ in which they grow and multiply producing daughter merozoites that upon release further invade naive erythrocytes, thus establishing the blood-stage life cycle of the parasite, which is responsible for all the clinical symptoms and pathology associated with malaria^9^. Therefore, erythrocyte invasion by *P. falciparum* merozoites is the most fundamental step that ensures parasite multiplication at levels that lead to the malarial disease. Importantly, *P. falciparum* erythrocyte invasion remains an attractive process to investigate in order to advance our understanding of the basic parasite biology as well as to translate the findings in the development of novel malaria intervention strategies. Merozoites are extracellular invasive forms of blood-stage parasites that are susceptible to host immune responses. In this regard, one of the major focuses of both vaccine and drug development targeting the erythrocyte invasion process is to induce either potent antibodies or small molecule inhibitors against key parasite ligands that efficiently block erythrocyte invasion by abrogating their attachment with the erythrocyte. However, *P. falciparum* erythrocyte invasion is a highly complex, multistep process that is facilitated by the sequential involvement of multiple ligand-receptor interactions during the distinct steps of invasion. These parasite ligands bind with specific receptors on the erythrocyte surface and are known to mediate steps such as initial attachment of the merozoite with the target erythrocyte till the formation of the junction. The molecular redundancy at the disposal of *P. falciparum* allows it to invade erythrocytes through multiple independent pathways even in the absence of one or two ligand-receptor interactions. The repertoire of invasion associated parasite molecules remains very large and has still not been completely elucidated or defined.

The *P. falciparum* genome is 26 MB in size and codes for around 5300 genes^7^,^8^,^9^. Approximately, 60% of the proteins encoded by these genes have no defined function associated with them and bear little or no similarity with proteins of other organisms^10^,^11^,^12^. About 2700 genes are expressed during the 48 hour blood-stage cycle with a significant number of hypothetical genes having a similar transcript expression profile matching with that of known invasion related genes^10^,^11^,^12^. Thus, there are still numerous hypothetical genes that may be involved in erythrocyte invasion. It is highly possible that like PfRH5, there may be more efficacious blood stage malaria vaccine candidates that remain to be identified. Thus, it is important to identify and characterize novel *P. falciparum* blood-stage proteins involved in erythrocyte invasion. These efforts not only augment our understanding of the basic biology of the parasite but also expand our repertoire of antigens that could induce potent invasion-inhibitory antibodies. Hence, to identify novel targets, it is important to study new parasite ligands that mediate the complex process of erythrocyte invasion.

Here, we report the functional characterization of a novel *P. falciparum* protein, (Plasmodb IDs: PF3D7_1012200, PF10_0119; GenBank accession: Q8IJS3) that is involved in the process of erythrocyte invasion by *P. falciparum* merozoites, which we have coined as ****P***. ***f***alciparum* **R**hoptry associated **A**dhesin (PfRA) based on its localization and erythrocyte binding function. Like other invasion related proteins, PfRA expression was observed only at the schizont stage of the intra-erythrocytic parasite across multiple *P. falciparum* strains. PfRA was observed to be localized in the apical organelle, rhoptry and undergo translocation to the merozoite surface during erythrocyte invasion. PfRA exhibits erythrocyte binding activity and acts as a ligand engaging with sialic acids on the erythrocyte surface, thus mediating invasion through the sialic acid dependent pathway. PfRA antibodies specifically blocked the erythrocyte binding of both native and recombinant PfRA, and inhibited merozoite invasion. In summary, we have identified and characterized a novel *P. falciparum* rhoptry adhesive protein involved in erythrocyte invasion.

## Results

### Identification of the *P. falciparum* Rhoptry associated Adhesin (PfRA)

*P. falciparum* invasion related proteins are known to be maximally expressed during the late schizont stages when the merozoites have developed and matured. Further, they are known to be either located on the merozoite surface or in the apical organelles (micronemes, rhoptries, dense granules) from where they are translocated to the surface. These proteins share some distinct features such as either the presence of a signal sequence that directs them through the secretory pathway to the parasite surface or a transmembrane domain/GPI-anchor that secures them on the merozoite surface.

Based on these guidelines, PfRA (Plasmodb IDs: PF3D7_1012200; PF10_0119; GenBank accession: Q8IJS3) was identified as part of our efforts to search the *P. falciparum* transcriptome databases for novel genes whose expression profile matched with that of well characterized genes known to play a role in *P. falciparum* erythrocyte invasion . Three *P. falciparum* expression profiling studies^10^,^11^,^12^ using transcriptomics had conclusively demonstrated that parasite gene transcripts were expressed in a well-orchestrated and regulated manner during the 48 hour blood-stage life cycle, of which genes involved in erythrocyte invasion are expressed only during the late schizont stages (40–48 hours).

**Figure 1:**
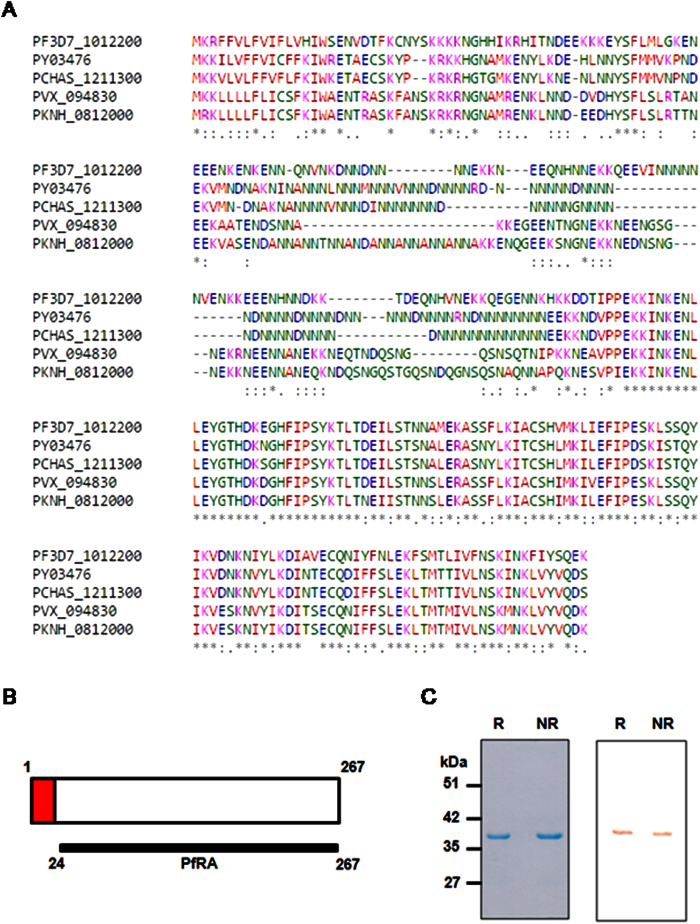
PfRA homologues in different *Plasmodium* species and generation of recombinant PfRA. (**A**) Alignment of PfRA protein sequences from different *Plasmodium* species, *P. vivax* strain Sal-1 (PVX_094830), *P. knowlesi* (PKNH_0812000), *P. yoelii* (PY03476) and *P. chabaudi* (PCHAS_1211300). “*” identical residues; “:” conserved substitutions; “.” semi-conserved substitutions. (**B**) Schematic representation of the 267 amino acid PfRA protein (Plasmodb ID: PF3D7_1012200). PfRA comprises of an N-terminal signal sequence (residues 1 to 22). The region (24–267) was expressed in a recombinant form and antibodies were raised against this region. **(C)** Purified recombinant PfRA (rPfRA) run on SDS-PAGE and detected by immunoblotting using anti-His tag antibodies. R: reducing; NR: Non-reducing.

One of the *P. falciparum* transcriptome profiling studies had assigned PfRA to be present in a cluster 15 that comprised of invasion associated proteins^10^, while another study predicted PfRA as a putative vaccine candidate on the basis of its expression profile matching with that of seven known blood-stage vaccine candidates^11^. The third expression profiling study also predicted a role for PfRA in erythrocyte invasion and further demonstrated that GFP-tagged PfRA was apically localized in both late schizont-stage parasites and free merozoites^12^. The apical localization of PfRA was consistent with the presence of an N-terminal signal sequence suggesting that the protein was secreted to the parasite surface. PfRA orthologues were also identified in *P. vivax* (PVX_094830), *P. knowlesi* (PKNH_0812000), *P. yoelii* (PY03476) and *P. chabaudi* (PCHAS_1211300) with a high degree of identity between residues 152 and 265 (Fig. 1A) suggesting that it played a putative conserved role across multiple *Plasmodium* species^12^. Further, PfRA was found to be highly conserved among both laboratory adapted clones (Supplementary Fig. S1) and field isolates (Supplementary Fig. S2) of *P. falciparum* that have origins from different worldwide locations (Supplementary Table S1, S2). Among nine laboratory adapted clones, only one polymorphism K136I was specifically observed in only the HB3 clone (Supplementary Fig. S1) with an identical PfRA sequence observed for all other 8 clones (Supplementary Fig. S1). In 57 field isolates primarily obtained from the African continent, the PfRA protein sequence displayed only two polymorphisms, K136I and A200S (Supplementary Fig. S2), substantiating PfRA as a highly conserved protein. Thus, based on the above rationale, PfRA was predicted to be a novel and highly conserved protein with a putative role in erythrocyte invasion that was selected to functionally validate its role during erythrocyte invasion.

### Expression of recombinant PfRA and generation of specific antibodies

The PfRA native parasite protein comprises of 267 amino acids with a putative N-terminal signal peptide (amino acid 1–22) but lacks any predicted membrane anchorage moiety (Fig. 1B). A 244 amino-acid sequence (Lys24-Lys267) of PfRA from the *P. falciparum* clone 3D7 was cloned in the T7 promoter based expression plasmid pET-24b (Novagen). The recombinant PfRA (rPfRA) protein was expressed in *E. coli* BL21-Codon Plus (DE3) cells with a C-terminal 6XHis tag. rPfRA was expressed in a soluble form in the cytosol and was further purified to homogeneity by immobilized metal affinity chromatography (Ni-NTA, G-Biosciences), followed by anion-exchange chromatography and size exclusion chromatography. rPfRA migrated as a single band on SDS-PAGE under both reducing and non-reducing conditions (Fig. 1C). rPfRA was detected in immunoblots using the anti-His tag antibody (Fig. 1C), confirming its expression with a C-terminal 6XHis tag. rPfRA was subjected to trypsin digestion followed by LC-MS (liquid chromatography-mass spectrometry) analysis (Orbitrap VELOS PRO; Thermo Fisher Scientific). The mass spectrometric analysis of rPfRA identified high scoring peptides corresponding to native PfRA confirming the identity of the recombinant protein (Supplementary Table S3).

In order to raise specific anti-PfRA antibodies, mice and rabbit were immunized with rPfRA using standard protocols described previously^13^. High titer antibodies against rPfRA were detected in both mice (end point 1:320,000) and rabbit (end point 1:640,000) (Supplementary Fig. S3). In immunoblots anti-PfRA antibodies readily detected rPfRA, whereas the corresponding pre-immune antibodies failed to do so, further confirming the specificity of the anti-PfRA antibodies (Supplementary Fig. S4).

### Expression of PfRA in asexual blood stage parasites

**Figure 2:**
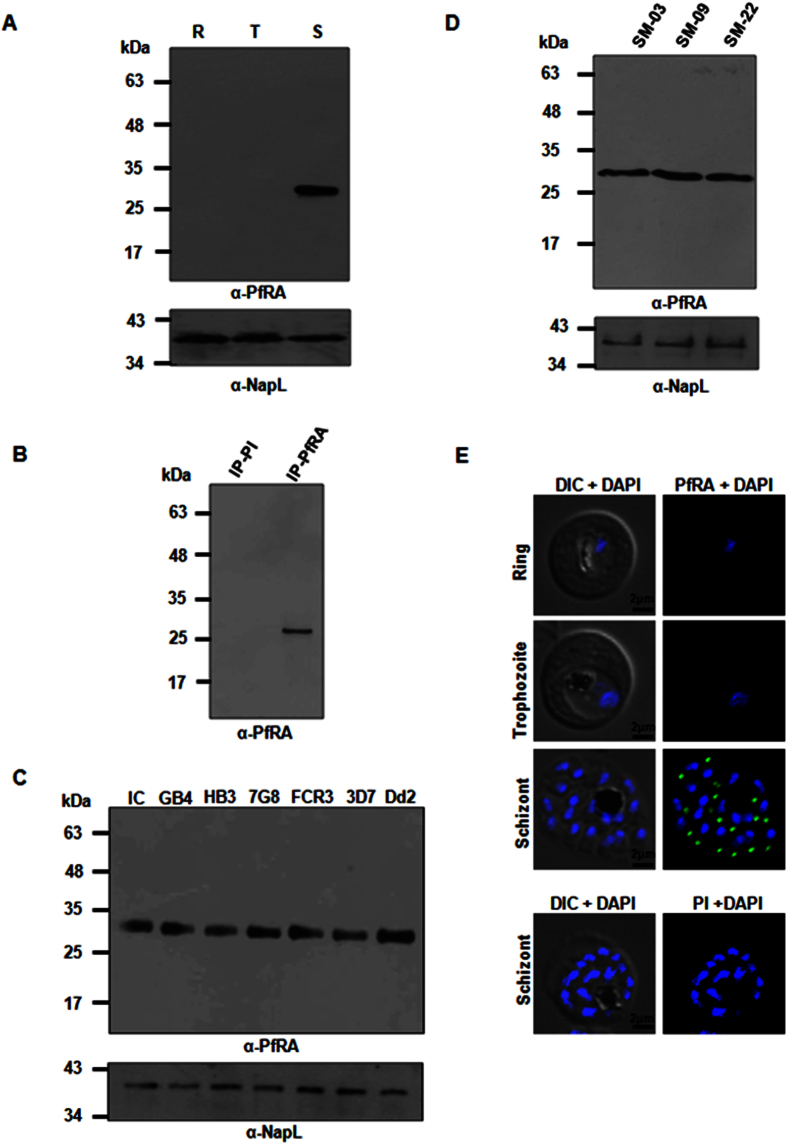
Native protein expression of PfRA in *P. falciparum*. (**A**) The 30 kDa PfRA parasite protein was detected by immunoblotting using specific PfRA mice antibodies only in the schizont stages of the intra-erythrocytic life cycle (R: ring; T: trophozoite; S: schizont). Expression of the nuclear protein, NapL was analyzed as a control. (**B**) The 30 kDa PfRA native parasite protein was immunoprecipitated by the PfRA mice antibodies and then detected by immunoblotting using PfRA rabbit antibodies. Pre-immune mice antibodies failed to immunoprecipitate the native PfRA parasite protein. **(C)** PfRA expression was detected in seven *P. falciparum* worldwide strains that differ in their invasion phenotypes. **(D**) PfRA expression was detected in three *P. falciparum* field isolates, SM-03, SM-09 and SM-22 that had undergone minimal *in vitro* cultivation. **(E)** Native PfRA expression was further analyzed by confocal immunofluorescence microscopy. Immunofluorescence assays were performed on fixed smears of different stages of the *P. falciparum* intra-erythrocytic life cycle. PfRA (green) staining was detected by the anti-PfRA mouse antibodies only in the schizont stages. No signal was observed in trophozoites and schizonts. A distinct punctate staining of PfRA was observed in the schizont which is characteristic of apically localized proteins. Pre-immune antibodies failed to detect any staining. Nuclei were stained with the DNA intercalating dye, DAPI. (Scale bar, 2 μm.)

To investigate the expression of PfRA during different stages of the intra-erythrocytic cycle, highly synchronized ring, trophozoite and schizont stage parasites of the *P. falciparum* 3D7 clone were harvested, saponin lysed and the total parasite proteins were obtained using detergent based extraction. Equal amount of the three protein samples were subjected to immunoblotting using specific anti-PfRA antibodies. The anti-PfRA antibodies specifically detected a ~30 kDa band corresponding to the expected size of native PfRA only at the schizont stage (Fig. 2A), while no proteins were detected during the ring and trophozoite stages. As a control, the *P. falciparum* nuclear protein NapL (nucleosome assembly protein L^14^) was detected equally among all the three stages (Fig. 2A). The specificity of the 30 kDa protein band was further confirmed by immunoprecipitation using the specific PfRA antibodies (Fig. 2B) that was followed by tryptic digestion and mass spectrometry. The mass spectrometric analysis identified a number of specific peptides (Supplementary Table S4) from native PfRA that confirmed the identity of the 30 kDa native parasite protein as *P. falciparum* Rhoptry associated Adhesin (PfRA; Pf3D7_1012200).

The expression of native PfRA was also analyzed by immunoblotting in seven *P. falciparum* laboratory adapted strains (3D7, 7G8, HB3, Dd2, Indochina, FCR3, GB4) with origins from different geographical regions of the world and three field isolates from Mozambique that underwent minimal *in vitro* cultivation (5–6 cycles). The 30 kDa PfRA was detected in all the seven laboratory adapted strains (Fig. 2C) as well as the three field isolates (Fig. 2D) that had undergone a few cycles of *in vitro* culture confirming that conservation of PfRA expression among worldwide *P. falciparum* strains was not selected under *in vitro* culture conditions . Expression of the *P. falciparum* nuclear protein, NapL^14^, was analyzed as a loading control among all the parasite stains (Fig. 2C,D).

The stage specific expression of PfRA during the different asexual stages of the parasite’s life cycle was further confirmed by confocal immunofluorescence microscopy. As observed in the immunoblots, immunofluorescence staining for PfRA was also detected exclusively in the schizont stage parasites (Fig. 2E). PfRA was observed to have an apical localization consistent with a previous report^12^. The corresponding pre-immune sera used as negative control did not yield any fluorescence signal in the schizont stage parasites. These results confirmed that native PfRA was expressed only during the late schizont stages of the intra-erythrocytic developmental cycle.

### PfRA is a rhoptry protein that is translocated to the apical merozoite surface during erythrocyte invasion

**Figure 3:**
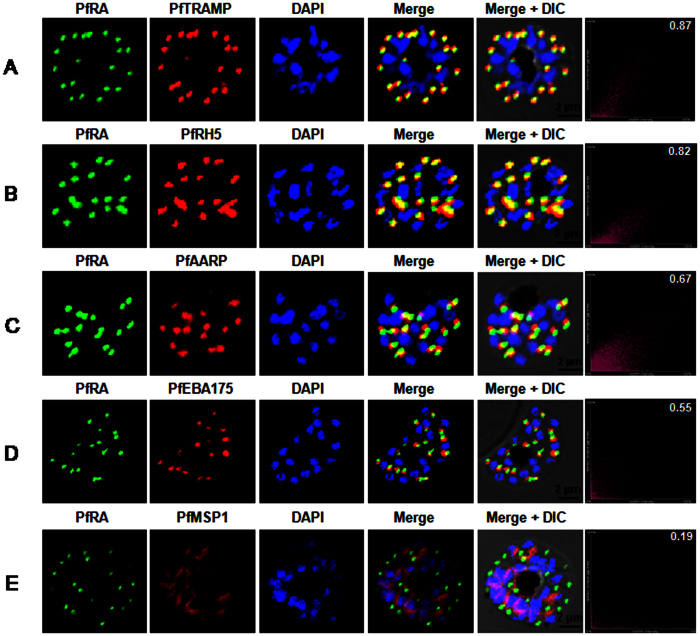
PfRA localizes to the rhoptries of *P. falciparum* schizonts and merozoites. Co-localization of PfRA with **(A)** PfTRAMP, **(B)** PfRH5, **(C)** PfAARP, **(D)** PfEBA-175 and **(E)** MSP-1 in schizont stage parasites by confocal immunoflourescence microscopy. PfRA (green) co-localizes with rhoptry bulb protein PfTRAMP (red) and PfRH5 (red) but does not co-localize with the micronemal protein PfEBA-175 (red) and merozoite surface protein MSP-1 in schizonts. PfRA (green) showed partial co-localization with rhoptry neck protein PfAARP (red). The co-localization of PfRA was quantitatively analyzed and the Pearson correlation co-efficient with PfTRAMP (0.87) and PfRH5 (0.82) suggested that the two proteins were co-localized with PfRA. The co-efficients of PfRA with PfAARP, PfEBA-175 and MSP1 were lower than 0.7 indicating that the three proteins were not co-localized with PfRA. Nuclei were stained with DNA intercalating dye DAPI. (Scale bar, 2 μm.)

To elucidate the sub-cellular localization of native PfRA, confocal immunofluorescence microscopy based co-localization studies were performed using antibodies against a repertoire of parasite proteins that are known to be located on merozoite surface (MSP-1)^15^ or inside different apical organelles (rhoptry bulb: PfRH5/PfTRAMP^16^,^17^,^18^; rhoptry neck: PfAARP^19^; microneme: EBA-175^20^). Co-immunostaining of PfRA with the surface marker protein MSP-1 in schizonts showed PfRA to be localized in the apical organelle and not on the merozoite surface (Fig. 3E). PfRA staining showed maximum overlap with PfTRAMP (Fig. 3A) and PfRH5 (Fig. 3B) suggesting PfRA to be a rhoptry bulb protein. PfRA did not co-localize with the micronemal protein EBA-175 (Fig. 3D) and showed partial co-localization with the rhoptry neck protein, PfAARP (Fig. 3C). Thus, consistent with the previous report^12^ that demonstrated apical localization of PfRA, we have shown PfRA to be localized in the apical organelle, rhoptry.

**Figure 4:**
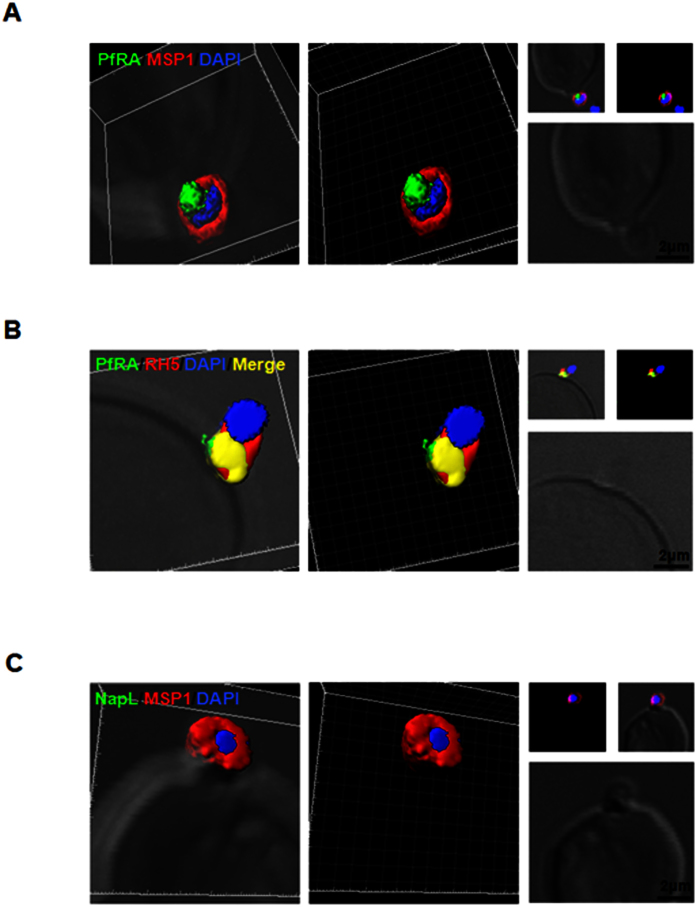
PfRA is present at the apical surface of an invading merozoite. Confocal immunofluorescence microscopy analysis of PfRA localization in cytochalasin-D treated invading merozoites under non-permeable fixing conditions using glutaraldehyde /paraformaldehyde. **(A)** 3D reconstruction of z-stacks during merozoite invasion co-immunostained with MSP-1and PfRA. PfRA detected at the apical end of the merozoite surface. In the 3D images, the γ settings were altered for visual representation only. **(B)** PfRA (green) co-localizes with rhoptry bulb marker PfRH5 (red) that is known to be translocated to the merozoite surface to engage with its erythrocyte receptor, Basigin. **(C)** As a control, staining of a nuclear parasite protein, NapL, was not detected under the same non-permeable fixing conditions confirming that the fluorescent signals were obtained only from surface localized proteins. Nuclei were stained with DNA intercalating dye DAPI (Scale bar, 2 μm.)

Most of the parasite proteins present in apical organelles (rhoptry, microneme) and implicated to play a role in invasion are secreted to the surface of an invading merozoite. Immunofluorescence staining of cytochalasin-D treated invading merozoites that were fixed using a non-permeable fixative, paraformaldehyde/glutaraldehyde^17^ showed PfRA to be apically localized on the merozoite surface (Fig. 4A), where it co-localized with the essential parasite ligand, PfRH5, also known to be localized in the rhoptry (Fig. 4B). As a control, staining for the nuclear parasite protein, NapL was not detected (Fig. 4C) as reported previously^17^ and confirmed the fixation under non-permeable conditions. Thus PfRA is a rhoptry bulb protein that is translocated to the apical surface of an invading merozoite during invasion, suggesting a role in erythrocyte invasion.

### PfRA binds to human erythrocytes in a sialic-acid dependent manner

**Figure 5:**
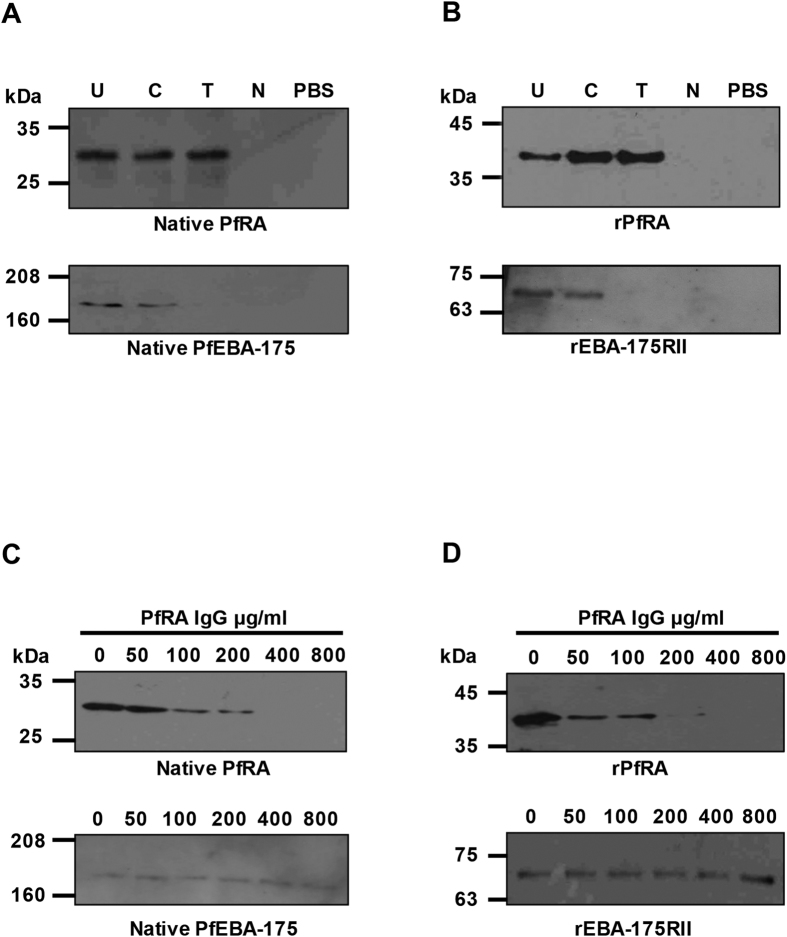
PfRA exhibits erythrocyte binding activity and anti-PfRA antibodies block erythrocyte binding of the native PfRA and rPfRA proteins. (**A**) Binding of native PfRA from the 3D7 culture supernatant with untreated and enzyme treated erythrocytes. Native PfRA bound untreated human erythrocytes (U), chymotrypsin treated human erythrocytes (**C**) and trypsin treated human erythrocytes (T), but failed to bind with neuraminidase treated human erythrocytes (N). Thus, PfRA binds human erythrocytes in a sialic acid-dependent, trypsin- and chymotrypsin-resistant, manner. (**B**) rPfRA bound human erythrocytes with the same specificity as that of native PfRA. Native PfEBA-175 and rPfEBA-175RII (recombinant receptor binding domain of PfEBA-175) were analyzed as a control with the same set of enzymatically treated erythrocytes. Both native PfEBA-175 and rPfEBA-175RII bind the sialoglycoprotein, Glycophorin A and failed to bind the neuraminidase and trypsin treated erythrocytes. PBS (pH 7.4) contained no protein. As a control, no specific protein was detected in the eluate fractions with Phosphate Buffer Saline (PBS) pH 7.4, suggesting that no non-specific erythrocyte protein was detected in the assay. Purified antibodies (total rabbit IgG) against recombinant rPfRA blocked erythrocyte binding of both the native PfRA (**C**) and rPfRA proteins (**D**) in a dose dependent manner. The PfRA antibodies had no effect on the erythrocyte binding of native PfEBA-175 or rPfEBA-175RII even at the maximum concentration of 800 μg ml^−1^ (total IgG).

Merozoite proteins involved in erythrocyte invasion are known to be shed into the culture medium (culture supernatant) during the invasion process^13^,^15^,^16^,^17^,^18^,^19^,^20^,^21^. The culture supernatant serves as a good source of soluble *P. falciparum* parasite proteins especially those involved in erythrocyte invasion. To determine the erythrocyte binding activity of native PfRA, standard erythrocyte binding assays were performed by incubating untreated and enzymatically (neuraminidase, trypsin, chymotrypsin) treated human erythrocytes with the parasite culture supernatant as reported earlier^21^,^22^. Native PfRA from parasite culture supernatant bound untreated as well as trypsin and chymotrypsin treated erythrocytes but failed to bind with neuraminidase treated erythrocytes (Fig. 5A) suggesting the erythrocyte binding phenotype of PfRA to be sialic acid dependent (neuraminidase sensitive), trypsin resistant and chymotrypsin resistant. Similar to native PfRA, rPfRA also bound human erythrocytes in a sialic acid dependent, trypsin resistant and chymotrypsin resistant manner (Fig. 5B). As a control, no non-specific bound proteins were detected when the human erythrocytes were incubated with only phosphate buffer saline (Fig. 5A,B), confirming the specific erythrocyte binding activity of both native and recombinant PfRA. Further, the erythrocyte binding activity of native PfEBA-175 was analyzed from the same culture supernatant sample along with that of recombinant PfEBA-175RII (Region II receptor-binding domain of PfEBA-175) as a control for the enzymatic treatments of the human erythrocytes. EBA-175 binds with the sialic acid residues of glycophorin A and is known to be sensitive to individual treatments of the erythrocytes with neuraminidase and trypsin^23^,^24^. On the other hand, glycophorin A is intact on the surface of chymotrypsin treated erythrocytes. Thus, as expected, both native PfEBA-175 and the rPfEBA-175RII protein bound with chymotrypsin treated erythrocytes but failed to bind neuraminidase or trypsin treated erythrocytes (Fig. 5A,B).

In addition to the standard procedure of analyzing erythrocyte binding activity through immunoblotting, the erythrocyte binding activity of native and recombinant PfRA was also analyzed by flow cytometry as reported previously for PfTRAMP^18^. Consistent with the immunoblotting data, the flow cytometry analysis also showed the erythrocyte binding phenotype of both native and recombinant PfRA to be neuraminidase sensitive, but trypsin and chymotrypsin resistant (Supplementary Fig. S5). Infact, flow cytometry is more quantitative and the binding of PfRA with trypsin and chymotrypsin treated erythrocytes was observed to be higher than that with untreated erythrocytes, suggesting that the enzymatic treatment augments the erythrocyte binding activity of the PfRA parasite protein.

### Anti-PfRA antibodies specifically abrogate the binding of both native and recombinant PfRA with human erythrocytes

Since both native and recombinant PfRA protein display specific erythrocyte binding activity, we analyzed the ability of anti-PfRA antibodies to specifically inhibit the erythrocyte binding activity of native and recombinant PfRA. Total IgGs purified from rabbit sera raised against rPfRA specifically blocked the binding of both native and recombinant PfRA proteins to human erythrocytes in a dose dependent manner (Fig. 5C,D). At a total IgG concentration of ≤400 μg ml^−1^, anti-PfRA IgGs completely blocked the binding of both native and recombinant PfRA; whereas even at a higher concentration of 800 μg ml^−1^ the anti-PfRA IgGs did not block the erythrocyte binding activity of PfEBA-175 or its recombinant Region II receptor-binding domain, PfEBA-175RII (Fig. 5C,D). These results clearly demonstrate that the anti-PfRA antibodies specifically recognize PfRA and abrogate its interaction with the erythrocyte surface.

### PfRA is membrane associated

**Figure 6:**
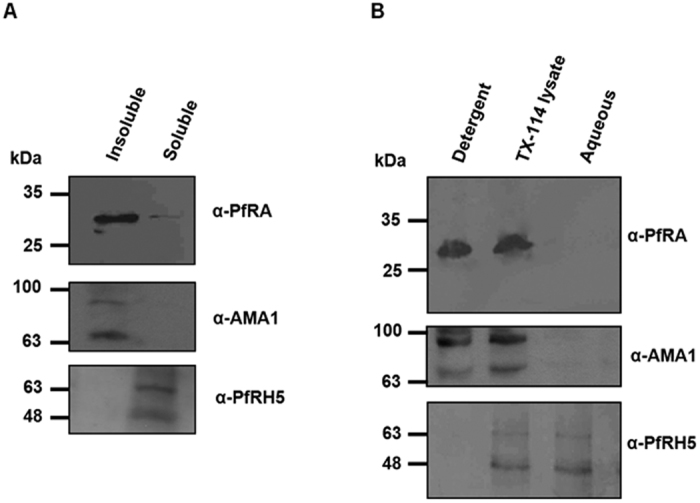
Solubility properties of PfRA. (**A**) Sodium carbonate extraction of the hypotonically lysed 3D7 schizont stage parasites produced soluble and insoluble fractions. Immunoblotting analysis detected PfRA in the insoluble fraction. AMA1, a Type I transmembrane protein was detected in the insoluble fraction compared to PfRH5 that lacks a transmembrane domain and is known to detected in a soluble form. **(B)** Triton X-114 phase partitioning of the schizont-stage lysate also showed PfRA and AMA-1 in the detergent insoluble fraction and PfRH5 in the aqueous fraction.

PfRA has a putative signal peptide at the N-terminus for entry into the secretory pathway through endoplasmic reticulum, but lacks a predicted membrane anchorage moiety. However, since PfRA is translocated to the apical surface of an invading merozoite and exhibits erythrocyte binding, we wanted to confirm whether it is tightly or peripherally associated with the plasma membrane. To validate whether PfRA is directly tethered to the merozoite membrane, we first performed sodium carbonate extraction^25^ followed by Triton X-114 phase partitioning^17^,^26^ of schizont-stage parasites. Treatment of the hypotonically lysed schizont pellet with sodium carbonate (Na_2_CO_3_) detected PfRA in the pellet fraction (Fig. 6A), suggesting PfRA to be membrane bound. As controls, AMA-1, a Type-I integral membrane protein^27^ and PfRH5, a peripheral membrane protein^17^ were detected in the pellet and soluble fractions respectively, confirming the specificity of the protein extraction and partitioning (Fig. 6A).

After Triton X-114 phase partitioning, PfRA was detected only in the detergent-resistant fraction (Fig. 6B), further substantiating PfRA to be tightly associated with the membrane. AMA-1 and PfRH5 were detected only in the insoluble (detergent resistant) and aqueous fractions as reported earlier^17^,^27^ (Fig. 6B). Thus, while PfRA was demonstrated to be tightly associated with the membrane, the absence of any predicted membrane anchorage moiety in PfRA suggested that PfRA might be associated with an unknown membrane protein tightly anchored to the merozoite surface. However, our PfRA immunoprecipitation experiments followed by mass-spectrometric analysis failed to consistently detect any interacting partner of PfRA (Supplementary Table S4). The elucidation of the mechanism of membrane anchorage of PfRA remains an important question that will require further investigations.

### Invasion Inhibitory activity of anti-PfRA antibodies

**Figure 7:**
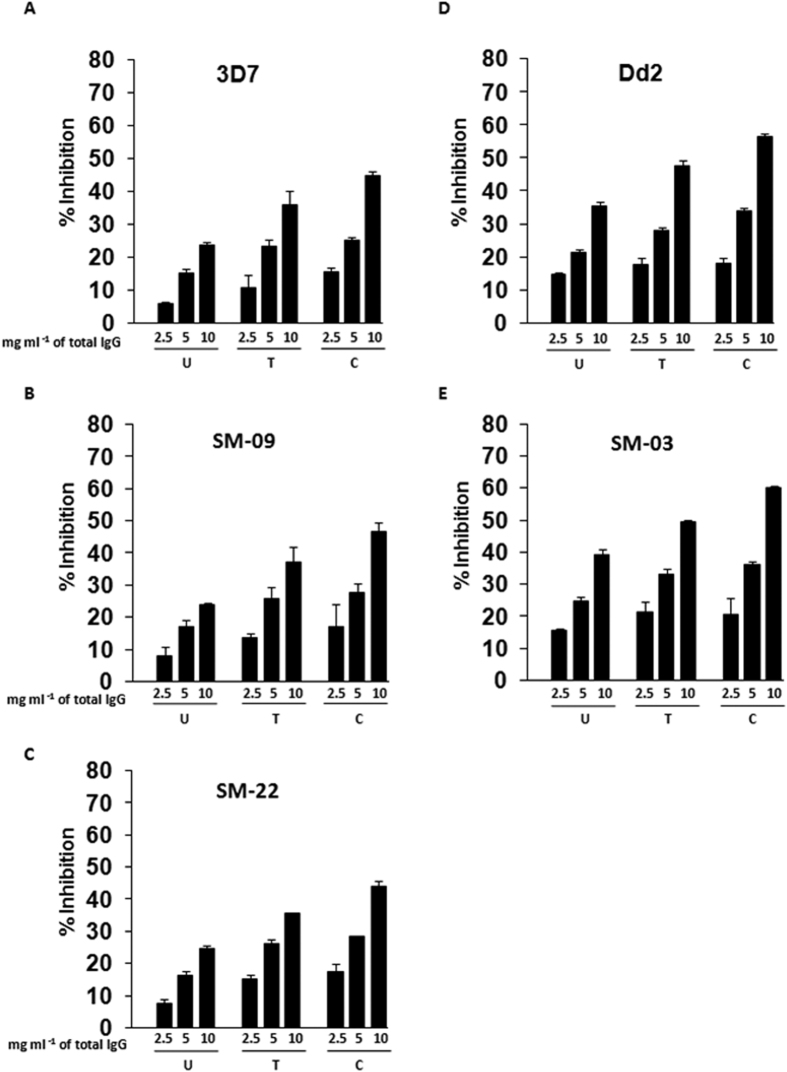
Invasion inhibitory activity of PfRA antibodies. Anti-PfRA rabbit antibodies were evaluated for their invasion inhibition activity against *P. falciparum* in untreated erythrocytes (U), trypsin-treated erythrocytes (T) and chymotrypsin-treated erythrocytes (**C**) against the sialic acid independent parasites, **(A)** 3D7 clone **(B)** SM-09 field isolate, **(C)** SM-22 field isolate; the sialic acid dependent parasites, **(D)** Dd2 clone and **(E)** SM-03 field isolate. The results against the laboratory adapted clones (3D7, Dd2) represent the average of three independent experiments performed in duplicate, whereas those against the field isolates (SM-09, SM-22, SM-03) represent the average of two independent experiments performed in triplicate. The error bars represent the standard error of the mean.

Purified total IgGs from rabbit sera raised against recombinant PfRA were analyzed for their inhibition of invasion into normal untreated (U), trypsin (T) and chymotrypsin (C) treated erythrocytes by *P. falciparum* strains 3D7 and Dd2 in standard invasion inhibition assays^28^. Anti-PfRA IgGs inhibited invasion of untreated erythrocytes by *P. falciparum* 3D7 in a dose dependent manner with ~24% inhibition observed at the highest concentration tested (10 mg ml^−1^) (Fig. 7A). Anti-PfRA IgGs (10 mg ml^−1^) inhibited invasion of trypsin treated and chymotrypsin treated erythrocytes by *P. falciparum* 3D7 by ~36% and ~45% respectively (Fig. 7A). The higher invasion inhibition of enzyme-treated erythrocytes suggests that PfRA plays a major role in the invasion of these enzyme-treated erythrocytes in which many receptors have been cleaved, leading to a restriction of ligand-receptor interactions and where PfRA binds with the residual sialic acid containing, trypsin and chymotrypsin resistant receptors. Similar invasion inhibition results were also observed against the *P. falciparum* clone, Dd2 (Fig. 7B) that is known to be sialic acid dependent and thus utilizes sialic acid binding ligands such as PfRA. Consistent with the detection of PfRA expression among the three *P. falciparum* field isolates that had undergone minimal cultivation, we analyzed the invasion inhibitory potential of the PfRA antibodies against the three field isolates (Fig. 7C–E). Two of the field isolates (SM09, SM22) had a sialic acid independent invasion phenotype (Supplementary Fig. S6), whereas the third (SM03) was sialic acid dependent (Supplementary Fig. S6). The invasion inhibitory activity of the PfRA antibodies was coherent with their activity observed against the laboratory-adapted clones. PfRA antibodies blocked invasion of SM09 and SM22 with an efficiency of 25% (10 mg/ml total IgG), which was augmented to 40–50% with enzymatic (trypsin/chymotrypsin) treatment of the target erythrocytes. On the other hand, like Dd2 the sialic acid dependent clone SM03 was inhibited with a higher efficiency of 40% (10 mg/ml) by the PfRA antibodies with normal untreated erythrocytes, which further increased to 50–60% with the enzymatically treated erythrocytes. The higher invasion inhibition by the PfRA IgGs against Dd2 and SM03 compared to 3D7, SM09 and SM22 is consistent with PfRA being a sialic acid binding parasite ligand that is highly utilized by the sialic acid dependent parasite strains.

**Figure 8:**
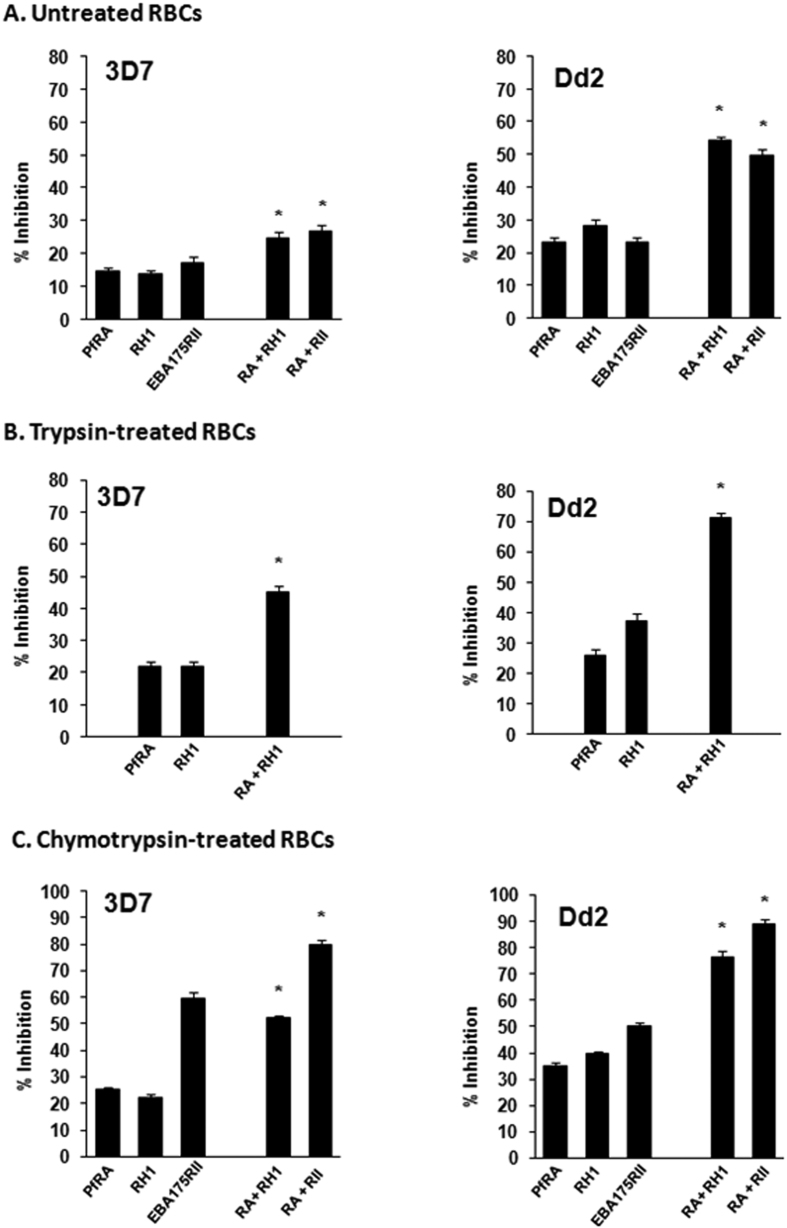
Invasion inhibitory efficacy of PfRA based antibody combinations against *P. falciparum*. Purified rabbit total IgG against the three individual antigens (PfRA, PfRH1, PfEBA-175RII) were evaluated individually (5 mg ml^−1^) as well as in dual combinations (5 mg ml^−1^ each; total 10 mg ml^−1^) against the sialic acid independent clone 3D7 and the sialic acid dependent clone Dd2 in **(A)** Untreated erythrocytes, **(B)** Trypsin-treated erythrocytes and **(C)** Chymotrypsin-treated erythrocytes. The results represent the average of three independent experiments performed in duplicate. The error bars represent the standard error of the mean. *denotes statistical significance between the invasion inhibition produced by the antibodies against the two antigen mixtures (PfRA + PfRH1, PfRA + PfEBA-175RII) with respect to the individual PfRA and PfRH1 antibodies and also among each other (p values < 0.05).

Anti-PfRA IgGs were also tested in combination with anti-PfRH1 and anti-PfEBA-175 rabbit IgGs for inhibition of invasion into untreated (U), trypsin (T) and chymotrypsin (C) treated erythrocytes by both *P. falciparum* clones 3D7 and Dd2. Individually, anti-PfRA IgGs (5 mg ml^−1^), anti-PfRH1 IgGs (5 mg ml^−1^) and anti-PfEBA-175RII IgGs (5 mg ml^−1^) inhibit invasion of untreated erythrocytes by *P. falciparum* 3D7 by 15%, 14%, and 17% respectively (Fig. 8A). The invasion inhibitory activity of the anti-PfRA IgGs (5 mg ml^−1^), anti-PfRH1 IgGs (5 mg ml^−1^) and anti-PfEBA-175RII IgGs (5 mg ml^−1^) against *P. falciparum* clone Dd2 were 23%, 29%, and 23% respectively (Fig. 8A). The antibody combinations of PfRA + PfRH1 IgGs (5 mg ml^−1^each) and PfRA + PfEBA-175RII inhibited erythrocyte invasion by *P. falciparum* 3D7 by 25% and 27%, respectively (Fig. 8A). The same set of antibodies individually and in combination blocked invasion of the *P. falciparum* Dd2 clone with a higher efficiency (Fig. 8A) consistent with Dd2 being a sialic acid dependent clone and all three antigens (PfRA, PfRH1, PfEBA-175) being sialic acid binding ligands.

Further, these antibodies individually and in combination exhibited a significantly higher invasion inhibitory activity against both *P. falciparum* 3D7 and Dd2 clones invading trypsin and chymotrypsin-treated erythrocytes. The combination of PfRA + PfRH1 IgGs (5 mg ml^−1^each) inhibited invasion of trypsin treated erythrocytes by the *P. falciparum* clones 3D7 and Dd2 with an efficiency of 45% and 71%, respectively (Fig. 8B). The combination of PfRA + PfEBA-175 RII could not be tested with trypsin treated erythrocytes as EBA-175 does not bind trypsin treated erythrocytes and thus is non-functional in their invasion.

The invasion inhibitory effect of individual PfRA antibodies was found to be maximum with chymotrypsin treated erythrocytes (Fig. 7A,B). A similar trend was observed with the PfRA based antibody combinations tested with chymotrypsin treated erythrocytes. The combination of PfRA + PfRH1 IgGs (5 mg ml^−1^each) inhibited invasion of chymotrypsin treated erythrocytes by the *P. falciparum* clones 3D7 and Dd2 with an efficiency of 52% and 77%, respectively (Fig. 8C). While, the combination of PfRA + PfEBA-175RII IgGs (5 mg ml^−1^each) inhibited invasion of chymotrypsin treated erythrocytes by the *P. falciparum* clones 3D7 and Dd2 with an efficiency of 80% and 89%, respectively (Fig. 8C). The high invasion inhibition of chymotrypsin treated erythrocytes is attributed to the significant role of EBA-175 in the invasion of chymotrypsin treated erythrocytes^29^, which is consistent with the high inhibition (3D7: 59%; Dd2: 50%) exhibited by individual PfEBA-175RII antibodies (5 mg ml^-1^) itself with chymotrypsin treated erythrocytes (Fig. 8C). The antibody combinations (PfRA + PfRH1; PfRA + PfEBA-175RII) elicited an invasion inhibition that was significantly higher than those observed for the individual antibodies (p < 0.05). Thus, the PfRA based antibody combinations appear to induce an additive inhibitory effect against these parasite strains substantiating that PfRA plays a role as a parasite ligand mediating erythrocyte attachment during the process of *P. falciarum* merozoite invasion

## Discussion

*P. falciparum* erythrocyte invasion is a complex, multistep process that is interwoven with a number of intricate molecular interactions^30^,^31^. While, our understanding of the molecular basis of erythrocyte invasion by *P. falciparum* has significantly advanced, it would be fair to say that we still do not have a complete understanding of this process. The *P. falciparum* genome is quite large and the parasite is known to utilize numerous parasite ligands at its disposal to mediate invasion through multiple redundant pathways. Among the 2800 genes expressed at the blood-stage, a large number remain uncharacterized with unknown functions^10^,^11^,^12^. With respect to the invasion process, several systems biology approaches have hinted that the total number of putative invasion related genes are quite high, of which the function are known only for few that includes MSPs, AMA-1, EBAs & PfRH proteins^10^,^11^,^12^. In this regard, efforts to identify novel parasite genes and characterize the role of their encoded proteins in erythrocyte invasion has major significance in elucidating the molecular basis of erythrocyte invasion.

Here, we have explored the subset of hypothetical genes that have been reported to be expressed during the late intra-erythrocytic stage of asexual parasites and based on published transcriptome data as well as bioinformatics analysis, we selected the novel gene PF3D7_1012200 (PF10_0119; Q8IJS3) to further investigate its potential role in erythrocyte invasion. Based on our results, we coined the name of the PF3D7_1012200 gene as *P. falciparum* Rhoptry associated Adhesin (PfRA). PfRA possesses an N-terminal hydrophobic signal peptide and was predicted to be secreted to the parasite surface. Importantly, it was conserved across different *Plasmodium* species suggesting a conserved and important role in erythrocyte invasion by all parasites. In order to functionally characterize this protein, we expressed full-length PfRA excluding the signal peptide as a recombinant protein and raised specific anti-PfRA antibodies. The antibodies detected native PfRA as a 30 kDa protein, expressed only during the schizont stage of the intra-erythrocytic parasite by immunoblotting and immunoprecipitation. The immunoprecipitated native protein (30 kDa) was further subjected to tryptic digestion followed by mass spectrometric analysis that confirmed the identity of the native parasite protein as PfRA. It is important to note that on SDS-PAGE, recombinant PfRA ran around 40 kDa whereas the native PfRA protein ran around 30 kDa. Both proteins were confirmed by mass spectrometric analysis to be PfRA. This discrepancy, which has been reported earlier for many other proteins^19^,^32^,^33^, could be explained by the presence of the 6xHis Tag that affects the mobility of the recombinant PfRA. Further, *P. falciparum* invasion related proteins are well known to be proteolytically processed and the native PfRA parasite protein might also be a processed form that runs at a lower molecular mass^34^,^35^,^36^,^37^,^38^. In fact, mass spectrometric (LC-MS) analysis of the immunoprecipitated native protein showed a lack of peptides from the N-terminal region (amino acids 23–97), with the residues 1–22 being the signal sequence, suggesting that native PfRA might be processed near the N-terminus.

The sequence of the native PfRA protein was found to be highly conserved among both laboratory adapted *P. falciparum* clones and strains isolated from several malaria endemic field sites with only two polymorphisms observed across the 267 amino acid protein. This observation along with the fact that PfRA homologues were observed in other *Plasmodium* species strongly suggest that PfRA has a conserved functional role. Furthermore, the expression of the native PfRA parasite protein was detected among seven worldwide *P. falciparum* laboratory adapted clones and three field isolates strains that underwent minimal *in vitro* cultivation, thus confirming that PfRA expression was not selected solely under *in vitro* culture conditions. In immunofluorescence confocal microscopy experiments, PfRA staining exhibited maximum co-localization with that of known rhoptry bulb proteins, PfRH5^17^ and PfTRAMP^18^, suggesting it to be a rhoptry bulb protein. Importantly, during erythrocyte invasion, PfRA was observed at the apical surface of the invading merozoite under non-permeabilized conditions. Thus, like other known invasion ligands^17^,^37^,^38^, PfRA is translocated from the rhoptry to the apical surface during merozoite invasion consistent with it playing a role in erythrocyte invasion. PfRA was observed to exhibit erythrocyte binding activity, thus acting as an adhesin that mediates attachment of the merozoite with the target erythrocyte. Both native and recombinant PfRA bound human erythrocytes in a sialic acid dependent manner with a phenotype that was sensitive to neuraminidase but resistant to either trypsin or chymotrypsin treatments. Similar studies in the recent past have identified primarily sialic acid independent ligands such as PfRH2^13^, PfRH4^21^, PfRH5 17,36,39, PfGAMA^35^ and PfMA^40^. In this context, our study has identified a novel sialic acid binding parasite ligand that adds to the existing repertoire of sialic acid dependent protein ligands such as EBA-175^24^, EBA-140^22^,^41^, EBA-181^42^ and PfRH1^43^. The erythrocyte binding activity of the recombinant PfRA matched with that of the native parasite protein suggesting that our recombinant protein was expressed with a structural integrity that matched that of the native protein. This was further exemplified by the observation that the anti-PfRA antibodies blocked the erythrocyte binding of the native and recombinant PfRA protein confirming their specificity.

The anti-PfRA antibodies exhibited invasion inhibitory activity consistent with the role of PfRA in erythrocyte attachment during invasion. PfRA antibodies (10 mg ml^−1^) blocked erythrocyte invasion by the sialic acid dependent strain Dd2 with an efficiency of 35% and the sialic acid independent strain, 3D7 with an efficiency of 24%. These invasion inhibition results are consistent with PfRA playing a role in a sialic acid dependent pathway and is similar to the invasion inhibition observed with antibodies against other sialic dependent parasite ligands such as PfEBA-175 and PfRH1^28^. This was further confirmed by the additive inhibition observed by antibody combinations including PfRA with either PfRH1 or PfEBA-175RII. The erythrocyte binding of PfRA, PfRH1 and PfEBA-175 was resistant to either trypsin or chymotrypsin treatments. Thus, the additive inhibitory effects of the antibody combinations were enhanced with trypsin and chymotrypsin treated erythrocytes in which the number of ligand-receptor interactions mediating invasion were restricted, further augmenting the role of the resistant parasite ligands. The invasion inhibition data further substantiates our observation of PfRA binding directly with host erythrocytes during invasion. The higher inhibition of PfRA antibodies observed with sialic acid dependent strains is consistent with their sole utilization of sialic acid binding ligands, which they achieve by downregulating the expression of sialic acid independent binding ligands such as RH2 and RH4^28^. Further, our results corroborates the approach of neutralizing the parasite by targeting multiple ligand-receptor interactions in combination.

Our goal was to identify novel merozoite antigens that play a role in erythrocyte invasion and also elicit potent invasion inhibitory antibodies that could potentially be taken forward for the development of new generation malaria vaccines. It is only through such initiatives that novel blood-stage vaccine candidates, PfRH5^39^ and CyRPA^17^ have been identified. Like several non-essential parasite ligands (EBA-175, PfRH1, PfRH2, PfRH4) that due to redundancy are unable to individually elicit potent invasion inhibitory antibodies, PfRA also does not fit the potential of a standalone vaccine candidate. However, a cocktail comprising of several merozoite antigens appears to be a feasible approach for developing a blood-stage malaria vaccine that would overcome the problem of redundancy. In this regard, several previous reports have demonstrated that antibodies against individual merozoite antigens (EBA-175, PfRH1, PfRH2, PfRH4) that do not exhibit individual potent strain-transcending invasion inhibition are able to produce potent additive or synergistic inhibition in combination^28^,^44^. These antibodies have been shown to even enhance the inhibitory activity of potent PfRH5 antibodies^36^,^45^. A crucial aspect of PfRA is its highly conserved nature that supports its potential use in a combination blood-stage vaccine.

An interesting aspect of PfRA is that it lacks any predicted membrane attachment moiety. However, sodium carbonate and Triton X-114 extraction experiments confirmed that PfRA is membrane associated. Our immunofluorescence imaging under non-permeabilized conditions also confirmed that PfRA is localized on the surface of the invading merozoite. In this regard, it appeared that PfRA might be interacting with some other parasite proteins that escort and anchor PfRA to the merozoite surface. However, based on our immunoprecipitation followed by mass spectrometry experiments, we did not find any other parasite protein interacting with PfRA. Thus, the mechanism through which PfRA is anchored on the merozoite surface warrants further investigation.

In summary, in this study we have identified and characterized a novel parasite ligand PfRA, which is localized in the rhoptries, undergoes translocation to the apical surface of an invading merozoite and mediates erythrocyte attachment by binding with a sialic acid dependent erythrocyte receptor. PfRA adds to the ever expanding repertoire of parasite ligands that constitute the large invasion machinery of the parasite and forms the basis for the enormous complexity that is reflected in the erythrocyte invasion process of *P. falciparum*.

## Methods

### Ethics statement

The animal studies described were approved by the International Centre for Genetic Engineering and Biotechnology (ICGEB) Institutional Animal Ethics Committee (IAEC; Reference Number MAL-89), according to the guidelines of the Department of Biotechnology, Government of India. The study protocol for the collection of *P. falciparum* field isolates by informed consent was approved by the National Ethics Review Committee of Mozambique and the Ethics Review Committee of the Hospital Clinic of Barcelona (Reference Numbers: 137/CNBS/05 and 200/CNBS/06).

### Cloning, expression and purification of the full-length recombinant PfRA

The DNA fragment corresponding to the full-length protein, PfRA (24–267 amino acids) excluding the N-terminal signal sequence was amplified using the primers: (Fwd–5′- TATGGAATTCcatatgAAATGTAATTATTCGAAGAAAAAA-3′; Rev– 5′-ATCGctcgagTTTT TCTTGTGAATAAATAAATTTG-3′) from cDNA, that had been prepared from 3D7 total RNA using the single-strand cDNA synthesis kit (Life technologies). The PCR product encoding rPfRA was digested with NdeI and XhoI (New England Biolabs, Beverly, MA) and inserted downstream of the T7 promoter in the *E. coli* expression vector, pET-24b (Novagen, San Diego, CA) with a C-terminal 6-histidine (6-His) tag to obtain the plasmid pPfRA-pET24b. The gene encoding PfRA got expressed in codon plus cells. Induction of bacteria cultures were done with 1 mM isopropyl-1-thio-β-D-galactopyranoside (IPTG) at an optical density of 1.0 at 600 nm. The cells were harvested by centrifugation at 4,000 g for 20 minutes after 4 hours post induction at 37 °C. For the purification of recombinant PfRA that got expressed in a soluble form in the bacterial cytosol, the total induced cell pellet was harvested by centrifugation at 4,000 g for 20 minutes and resuspended in lysis buffer (50 mM NaH2PO4, 300 mM NaCl, 20 mM Imidazole, pH 8.0 containing 0.5 mg/ml lysozyme and protease inhibitors). The recombinant PfRA protein was purified from the lyzed supernatant by immobilized metal affinity chromatography (IMAC) using the Ni-NTA resin (G Biosciences). Briefly, the Ni-NTA column was pre-equilibrated with the lysis buffer (10 column volumes) and the supernatant collected after cell lysis was loaded onto the column at a flow rate of 1 ml/min. After loading was complete, the resin was washed with 10 column volumes of wash buffer (50 mM NaH_2_PO_4_, 300 mM NaCl, 20 mM imidazole, pH 8.0) and the bound protein was eluted in 4 column volumes of elution buffer (50 mM NaH_2_PO_4_, 300 mM NaCl, 250 mM imidazole, pH 8.0).

These Ni-NTA purified fractions were analyzed by SDS–PAGE and the purified elutes were pooled, concentrated and further purified to homogeneity by anion exchange chromatography and size exclusion chromatography. The identity and purity of recombinant rPfRA protein was characterized by SDS-PAGE, Western Blotting, and liquid chromatography-mass spectrometry (LC-MS) analysis (Orbitrap VELOS PRO; Thermo Fisher Scientific).

### Parasite culture

The laboratory-adapted parasite strains were cultured *in vitro* according to methods described previously^46^,^47^. The parasites were grown at 4% hematocrit in human O + erythrocytes and RPMI 1640 medium supplemented with 0.5% Albumax, 24 mM HEPES, 360 μM hypoxanthine, 24 mM sodium bicarbonate and 10 μg/ml gentamicin at 37 °C with 5% CO_2_, 5% O_2_, 90% N_2_. Normal human erythrocytes (O+) were obtained from the Rotary Blood Bank in New Delhi, India. The serum and leukocytes were removed, and the erythrocytes were washed with modified RPMI 1640 medium, as mentioned above, with no supplement of Albumax.

The three *P. falciparum* field isolates were collected from Mozambican children (less than 5 years) at the Manhiça District Hospital (South Mozambique)^48^ and were further cultured for 5–6 cycles (10–12 days) at 2% hematocrit in human O + erythrocytes in a RPMI 1640 medium comprising containing 24 mM HEPES, 360 μM hypoxanthine, 2 g/L sodium bicarbonate, 5 g/L Albumax II, 3 g/L Glucose and 10 μg/L of gentamicin at 37 °C with 5% CO_2_, 5% O_2_, 90% N_2_.

### Animal Immunization and antibody purification

Specific antibodies were raised against the PfRA full-length protein by immunizing mice and rabbit. Briefly, mice and rabbit were immunized on day 0 with 25 μg and 100 μg of the protein respectively emulsified with complete Freund’s adjuvant (Sigma, St. Louis, MO). The animals were given two booster doses on day 28 and day 56, with the same amount of protein emulsified with incomplete Freunds’ adjuvant. Terminal bleeds were collected on Day 70. Sera were tested for antibody titers and the specific recognition of each recombinant protein by an enzyme-linked immunosorbent assay (ELISA).

Total IgG was purified from rabbit sera by using a protein G affinity column (GE Healthcare, Uppsala, Sweden), in accordance with the manufacturer’s instructions. The purified IgGs were dialyzed with RPMI and used in invasion inhibition assays as described earlier^28^. As an adjuvant negative control, we also had raised antibodies in mice, and rabbit against a non-related peptide (KESRAKKFQRKHITNTRDVD) from human pancreatic RNase that was also formulated with the same adjuvant (CFA/IFA) and injected in animals with the same schedule used for raising the antibodies against the recombinant proteins as described above.

### Immuno-blot analysis of parasite proteins

Synchronized *P. falciparum* blood stage parasites were harvested by centrifugation at ring, trophozoite and schizont stages, lysed with saponin (0.05%), washed three times with PBS. Pellets were lysed in RIPA buffer (150 mM NaCl, 1% NP-40, 0.5% sodium deoxycholate, 0.1% SDS, and 50 mM Tris, pH 8.0) with 1X protease inhibitor cocktail (Roche, USA) for two hours on ice with intermittent mixing. The lysed material was spun at 16000 g for 30 minutes. The clear supernatant (parasite lysate) was collected and total protein concentration was measured using BCA kit (Pierce, USA). 0.15 mg of total protein from each stage was then boiled in sample loading buffer and separated on 12% SDS-PAGE gel. The resolved proteins were transferred to 0.45 mm nitrocellulose membrane, blocked in 5% skimmed milk in PBS overnight at 4 °C and probed with anti-PfRA mouse sera at 1:250 dilution for one hour. The blots were then washed, probed for 1 hour with the corresponding HRP-conjugated secondary antibodies. The blots were developed using Western lightning plus ECL reagent (Perkin Elmer). Immunoblotting for different *P. falciparum* strains and field isolates was done in a similar manner.

### Immunofluorescence assay (IFA)

Ring, trophozoite and schizont stage parasites from 3D7 *P. falciparum* clone were smeared on glass slides, air dried and fixed with pre-chilled methanol (−20 °C) for 30 minutes. Fixed slides were air dried and blocked with 3% BSA in PBS for 2 hours at room temperature (~25 °C). The slides were washed with PBST (PBS containing 0.05% Tween-20) and PBS. For PfRA co-localization studies slides were probed with anti-PfRA mice sera (1:100), anti-PfTRAMP rabbit sera (1:50), anti-EBA-175 rabbit sera (1:50), anti-PfAARP rabbit sera (1:100), anti-MSP-1 rabbit sera (1:50) and anti-PfRH5 rabbit sera (1:50). The slides were incubated with the respective primary antibodies for 1 hour at room temperature. Slides were washed with PBST and incubated with the corresponding Alexa Fluor 488/Alexa Fluor 594 conjugated secondary antibodies for 1 hour. The slides were then washed and mounted in ProLong Gold antifade reagent with 4′,6′-diamidino-2-phenylindole (Invitrogen) and were viewed on a super resolution confocal microscope (N-SIM, Nikon, Japan). The images were processed using Nikon NIS Elements AR 4.13.04.

### Isolation of Invasive merozoites and Immunofluorescence analysis

Merozoites were prepared using protocols reported earlier^17^. Briefly, synchronized mature segmented schizont stage parasites were allowed to rupture. The free merozoites were separated from the erythrocytes by centrifuging at 2000 g for 5 minutes. The supernatant collected were then spun at 4000 g for 5 minutes to pellet the merozoites. The merozoites were then treated with 1 μM of Cytochalasin D (Sigma Aldrich) for 5 minutes and then incubated for 10 minutes with fresh human erythrocytes. The sample was then washed twice with PBS to remove the free merozoites and then fixed with 0.0075% glutaraldehyde/4% (wt/vol) paraformaldehyde for 30 minutes at 4 °C. Immunofluorescence staining was performed as has been described earlier. The images were viewed on a super resolution confocal microscope (N-SIM, Nikon, Japan). The images were processed using Nikon NIS Elements AR 4.13.04. Three-dimensional reconstruction of the deconvolved Z-stacks were done using IMARIS as reported earlier^17^,^49^.

### Enzymatic treatment of erythrocytes and erythrocyte binding assays

Enzymatic treatment of erythrocyte with trypsin, chymotrypsin and neuraminidase were done as reported earlier^21^,^22^. Erythrocyte binding assays were performed using recombinant proteins or culture supernatants of *P. falciparum* 3D7 schizont infected erythrocytes as described previously^21^,^22^. Briefly, culture supernatant or recombinant protein was incubated with human erythrocytes at 37 °C following which the suspension was centrifuged through dibutyl phthalate. The supernatant and oil were removed by aspiration. Bound parasite proteins were eluted from the erythrocytes with 1.5 M NaCl. The eluted fractions were analyzed for the presence of PfRA by immunoblotting using anti-PfRA mouse sera (1:250). For antibody mediated blockade of erythrocyte binding assays, either culture supernatant (1 ml) or the corresponding recombinant protein (1 μg) was incubated with human erythrocytes in the presence of total IgG purified from the rabbit sera immunized with recombinant PfRA.

### Western Blot Analysis of the Immunoprecipitated Elutes

Both the immune as well as the corresponding pre-immune sera were used to perform the immunoprecipitation experiments. Elutes were run on SDS/PAGE and transferred to a nitrocellulose membrane. Immunoblot analysis was performed using antibodies from a distinct species than that used for immunoprecipitation.

### Carbonate extraction

Carbonate extraction of schizont-stage parasites were done as described earlier^25^. Briefly, schizont stage parasites were hypotonically lysed to remove the cytoplasmic fraction and the resultant membrane pellet was further incubated with 100 mM sodium carbonate, pH 11.5 on ice for 2 hr. The sample was centrifuged to obtain a soluble and insoluble pellet fraction. The insoluble fraction was further washed twice with PBS, denatured with 2X SDS loading dye. Both the soluble and insoluble fractions were analyzed by immunoblotting.

### Triton X-114 partitioning experiments

Triton X-114 partitioning experiments were done as described earlier^17^,^26^. Briefly, schizont stage parasites were lysed with1% Triton X-114 in PBS, spun and the supernatant was layered over ice-cold 6% sucrose and incubated at 37 °C for 5 minutes. The solution was spun (500 g) for 5 minutes at 37 °C. The aqueous and the pellet fraction were collected, washed, mixed with 2X SDS running dye and analyzed by immunoblotting.

### Invasion Inhibitory assay

One cycle FACS-based invasion inhibition assays using anti-PfRA total rabbit IgGs were performed as described previously^13^,^17^,^28^,^36^ against the sialic acid independent clone 3D7 and the sialic acid dependent clone Dd2 in untreated erythrocytes (U), trypsin-treated erythrocytes (T) and chymotrypsin-treated erythrocytes (C). For the three field isolates, two independent invasion assays were performed with parasites that had undergone *in vitro* cultivation for 5 cycles (10 days) and 6 cycles (12 days). The percent invasion inhibition for each immune IgG was calculated with respect to the control pre-immune IgG from the same animal. As another negative control, we used immune IgG raised against a nonrelated peptide from human pancreatic RNase that was also immunized with the same adjuvant (CFA or IFA) as used for raising the PfRA antibodies. The results against the laboratory clones represent the average of three independent experiments performed in duplicate, whereas those against the field isolates represent the average of two independent experiments performed in triplicate. The error bars represent the standard error of the mean. Statistical significance was calculated using the Student’s t*-* test (Graph Pad Prism software, version 6.03). *P* values of < 0.05 were considered statistically significant.

## Additional Information

**How to cite this article**: Anand, G. *et al.* A novel *Plasmodium falciparum* rhoptry associated adhesin mediates erythrocyte invasion through the sialic-acid dependent pathway. *Sci. Rep.* **6**, 29185; doi: 10.1038/srep29185 (2016).

## Supplementary Material

Supplementary Information
