# Supplementary material for: A novel Plasmodium falciparum rhoptry associated adhesin mediates erythrocyte invasion through the sialic-acid dependent pathway
Source: Sci Rep. 2016 Jul 7;6:29185. doi: 10.1038/srep29185 (PMC4935899; doi:10.1038/srep29185)
Supplement: Supplementary Information [file srep29185-s1.pdf]

Supplementary Information

A novel *Plasmodium falciparum* rhoptry associated adhesin mediates erythrocyte invasion through the sialic-acid dependent pathway

Gaurav Anand[1], K. Sony Reddy[1], Alok Kumar Pandey[1], Syed Yusuf Mian[2], Hina Singh[1], Shivani Arora Mittal[1], Emmanuel Amlabu[1], Quique Bassat[3,4], Alfredo Mayor[3,4], Virander Singh Chauhan[1], Deepak Gaur[2]*

[1]Malaria Group, International Centre for Genetic Engineering and Biotechnology (ICGEB), New Delhi, India.

[2]Laboratory of Malaria and Vaccine Research, School of Biotechnology, Jawaharlal Nehru University, New Delhi, India

[3]ISGlobal, Barcelona Ctr. Int. Health Res. (CRESIB), Hospital Clínic - Universitat de Barcelona, Barcelona, Spain

[4]Centro de Investigação em Saude de Manhiça (CISM), Maputo, Mozambique.

*Corresponding Author

Address correspondence to:

Deepak Gaur, Ph.D.
Laboratory of Malaria & Vaccine Research
School of Biotechnology
Jawaharlal Nehru University
New Delhi 110067 India
Tel: +91-9818807105
Fax: +91-11-26742040
E-mail: deepakgaur189@gmail.com

Supplementary Methods

Mass Spectrometric analysis of purified recombinant protein

In-gel digestion of the purified recombinant protein band was done to ascertain its identity. The corresponding protein bands from the coomassie stained SDS-PAGE gel were excised, reduced, and alkylated. Proteins were digested overnight with trypsin (Promega) and the corresponding peptides were eluted from the gel using trifluoroacetic acid. Mass spectrometry analysis and protein identification was done as described previously[1]

ELISA

Antibody responses in mice and rabbit were quantified by ELISA. Briefly, 96-well plates (Costar) were coated overnight with 0.2 μg per well of the recombinant protein in 0.06 M carbonate-bicarbonate buffer, pH 9.6 (Sigma). The plates were washed thrice with 0.05% Tween in PBS and further blocked with 2% skimmed milk in phosphate buffered saline (PBS) at 37°C for 2 h. Washing was sequentially repeated and serial dilutions of the primary sera (1:1,000-fold onwards) were prepared and incubated in the respective wells for 1 hour. The ELISA plate was subjected to stringent washing with PBS containing 0.05% Tween-20 and finally with PBS alone. Thereafter, a 1:10,000 dilution of the horseradish peroxidase conjugated secondary antibody (Sigma, St. Louis, MO) was added to each well and incubated for 60 minutes at 37°C. The enzymatic reaction was developed by the addition of *o*-phenylenediamine dihydrochloride (OPD) and hydrogen peroxide for 25 minutes at 37°C. The reaction was terminated by the addition of 2M sulphuric acid, and OD_{492} was recorded by using an ELISA microplate reader (Molecular Devices). Pre-immune (pre-bleed; PI) sera were used at similar dilutions as a control.

FACS-based erythrocyte binding assay (EBA)

Erythrocytes were incubated with 0.5 ml of culture supernatant at 37°C for 3 hours. After incubation, the erythrocytes were washed with PBS and bound PfRA on surface of erythrocytes was detected using anti-PfRA rabbit sera and Alexa 488 conjugated goat anti-rabbit IgG antibodies and were read on a FACS Caliber flow cytometer. The resulting flow cytometry data were analyzed using Cell Quest software.

Co-Immunoprecipitation and Mass Spectrometric Analysis

Immunoprecipitation experiments using lysate of the schizont stage parasites were performed as prescribed (Thermo scientific). The trypsin digested samples were analyzed on a nano-LC equipped Orbitrap VELOS PRO (Thermo Fisher Scientific) mass spectrometer as described previously[1]. The proteins were identified by blasting the peptides over a *Plasmodium falciparum* database (Uniprot), using proteome discoverer (Thermofisher) using standardized procedure as described earlier[1].

Invasion assays

Invasion assays were done as described previously[2]. Briefly, the parasites were first synchronized by the purification of schizont-stage (40-42 h) parasites on a percoll gradient. 0.4 million schizont stage percoll gradient purified parasites were incubated with 20 million uninfected normal as well as enzymatically treated erythrocytes at 2% haematocrit. After about 40 h post invasion, the parasite infected erythrocytes were stained with ethidium bromide dye and measured by a fluorescence activated cell sorter (FACS)-based assay. Relative invasion in enzyme treated erythrocytes was calculated with respect to the invasion observed in untreated erythrocytes. Two different experiments were performed in triplicate. For each assay Dd2 was

used as control for neuraminidase treated erythrocytes[2]. The error bars represent the standard error of the mean.

Sequence polymorphism analysis

To check sequence polymorphisms in the PfRA gene, available nucleotide sequences for the *P. falciparum* laboratory clones and field isolates was retrieved from the Plasmodb database, translated using ExPASy translate tool and aligned using multiple sequence alignment program Clustal Omega.

References

1. Reddy, K. S. *et al*. Multiprotein complex between the GPIanchored CyRPA with PfRH5 and PfRipr is crucial for *Plasmodium falciparum* erythrocyte invasion. *Proc. Natl. Acad. Sci. U.S.A.* **112**, 1179–1184 (2015).

2. Gaur, D., Storry, J.R., Reid, M.E., Barnwell, J.W., Miller, L.H. *Plasmodium falciparum* Is Able To Invade Erythrocytes through a Trypsin-Resistant Pathway Independent of Glycophorin B. *Infect Immun.* **71**, 6742-6746 (2003).

Supplementary Figure S1: Alignment of PfRA protein sequences from 9 *P. falciparum* laboratory strains (Clustal omega).

```
             1                                                          60
3D7          MKRFFVLFVIFLVHIWSENVDTFKCNYSKKKKNGHHIKRHITNDEEKKKEYSFLMLGKEN
Dd2-1        MKRFFVLFVIFLVHIWSENVDTFKCNYSKKKKNGHHIKRHITNDEEKKKEYSFLMLGKEN
T9_94        MKRFFVLFVIFLVHIWSENVDTFKCNYSKKKKNGHHIKRHITNDEEKKKEYSFLMLGKEN
Dd2-2        MKRFFVLFVIFLVHIWSENVDTFKCNYSKKKKNGHHIKRHITNDEEKKKEYSFLMLGKEN
7G8          MKRFFVLFVIFLVHIWSENVDTFKCNYSKKKKNGHHIKRHITNDEEKKKEYSFLMLGKEN
IT           MKRFFVLFVIFLVHIWSENVDTFKCNYSKKKKNGHHIKRHITNDEEKKKEYSFLMLGKEN
707A         MKRFFVLFVIFLVHIWSENVDTFKCNYSKKKKNGHHIKRHITNDEEKKKEYSFLMLGKEN
HB3          MKRFFVLFVIFLVHIWSENVDTFKCNYSKKKKNGHHIKRHITNDEEKKKEYSFLMLGKEN
CS2          MKRFFVLFVIFLVHIWSENVDTFKCNYSKKKKNGHHIKRHITNDEEKKKEYSFLMLGKEN
             ************************************************************

             61                                                        120
3D7          EEENKENKENNQNVNKDNNDNNNNEKKNEEQNHNNEKKQEEVINNNNNNVENKKEEENHN
Dd2-1        EEENKENKENNQNVNKDNNDNNNNEKKNEEQNHNNEKKQEEVINNNNNNVENKKEEENHN
T9_94        EEENKENKENNQNVNKDNNDNNNNEKKNEEQNHNNEKKQEEVINNNNNNVENKKEEENHN
Dd2-2        EEENKENKENNQNVNKDNNDNNNNEKKNEEQNHNNEKKQEEVINNNNNNVENKKEEENHN
7G8          EEENKENKENNQNVNKDNNDNNNNEKKNEEQNHNNEKKQEEVINNNNNNVENKKEEENHN
IT           EEENKENKENNQNVNKDNNDNNNNEKKNEEQNHNNEKKQEEVINNNNNNVENKKEEENHN
707A         EEENKENKENNQNVNKDNNDNNNNEKKNEEQNHNNEKKQEEVINNNNNNVENKKEEENHN
HB3          EEENKENKENNQNVNKDNNDNNNNEKKNEEQNHNNEKKQEEVINNNNNNVENKKEEENHN
CS2          EEENKENKENNQNVNKDNNDNNNNEKKNEEQNHNNEKKQEEVINNNNNNVENKKEEENHN
             ************************************************************

             121                                                       180
3D7          NDKKTDEQNHVNEKKQEGENNKHKKDDTIPPEKKINKENLLEYGTHDKEGHFIPSYKTLT
Dd2-1        NDKKTDEQNHVNEKKQEGENNKHKKDDTIPPEKKINKENLLEYGTHDKEGHFIPSYKTLT
T9_94        NDKKTDEQNHVNEKKQEGENNKHKKDDTIPPEKKINKENLLEYGTHDKEGHFIPSYKTLT
Dd2-2        NDKKTDEQNHVNEKKQEGENNKHKKDDTIPPEKKINKENLLEYGTHDKEGHFIPSYKTLT
7G8          NDKKTDEQNHVNEKKQEGENNKHKKDDTIPPEKKINKENLLEYGTHDKEGHFIPSYKTLT
IT           NDKKTDEQNHVNEKKQEGENNKHKKDDTIPPEKKINKENLLEYGTHDKEGHFIPSYKTLT
707A         NDKKTDEQNHVNEKKQEGENNKHKKDDTIPPEKKINKENLLEYGTHDKEGHFIPSYKTLT
HB3          NDKKTDEQNHVNEKIQEGENNKHKKDDTIPPEKKINKENLLEYGTHDKEGHFIPSYKTLT
CS2          NDKKTDEQNHVNEKKQEGENNKHKKDDTIPPEKKINKENLLEYGTHDKEGHFIPSYKTLT
             ************** *********************************************

             181                                                       240
3D7          DEILSTNNAMEKASSFLKIACSHVMKLIEFIPESKLSSQYIKVDNKNIYLKDIAVECQNI
Dd2-1        DEILSTNNAMEKASSFLKIACSHVMKLIEFIPESKLSSQYIKVDNKNIYLKDIAVECQNI
T9_94        DEILSTNNAMEKASSFLKIACSHVMKLIEFIPESKLSSQYIKVDNKNIYLKDIAVECQNI
Dd2-2        DEILSTNNAMEKASSFLKIACSHVMKLIEFIPESKLSSQYIKVDNKNIYLKDIAVECQNI
7G8          DEILSTNNAMEKASSFLKIACSHVMKLIEFIPESKLSSQYIKVDNKNIYLKDIAVECQNI
IT           DEILSTNNAMEKASSFLKIACSHVMKLIEFIPESKLSSQYIKVDNKNIYLKDIAVECQNI
707A         DEILSTNNAMEKASSFLKIACSHVMKLIEFIPESKLSSQYIKVDNKNIYLKDIAVECQNI
HB3          DEILSTNNAMEKASSFLKIACSHVMKLIEFIPESKLSSQYIKVDNKNIYLKDIAVECQNI
CS2          DEILSTNNAMEKASSFLKIACSHVMKLIEFIPESKLSSQYIKVDNKNIYLKDIAVECQNI
             ************************************************************

             241                        267
3D7          YFNLEKFSMTLIVFNSKINKFIYSQEK
Dd2-1        YFNLEKFSMTLIVFNSKINKFIYSQEK
T9_94        YFNLEKFSMTLIVFNSKINKFIYSQEK
Dd2-2        YFNLEKFSMTLIVFNSKINKFIYSQEK
7G8          YFNLEKFSMTLIVFNSKINKFIYSQEK
IT           YFNLEKFSMTLIVFNSKINKFIYSQEK
707A         YFNLEKFSMTLIVFNSKINKFIYSQEK
HB3          YFNLEKFSMTLIVFNSKINKFIYSQEK
CS2          YFNLEKFSMTLIVFNSKINKFIYSQEK
             ***************************
```

Supplementary Figure S2: Alignment of PfRA protein sequences from 57 *P. falciparum* field isolates (Clustal omega).

```
                1                                                          60
3D7             MKRFFVLFVIFLVHIWSENVDTFKCNYSKKKKNGHHIKRHITNDEEKKKEYSFLMLGKEN
TRIPS_480       MKRFFVLFVIFLVHIWSENVDTFKCNYSKKKKNGHHIKRHITNDEEKKKEYSFLMLGKEN
RV_3703         MKRFFVLFVIFLVHIWSENVDTFKCNYSKKKKNGHHIKRHITNDEEKKKEYSFLMLGKEN
RV_3637         MKRFFVLFVIFLVHIWSENVDTFKCNYSKKKKNGHHIKRHITNDEEKKKEYSFLMLGKEN
RV_3729         MKRFFVLFVIFLVHIWSENVDTFKCNYSKKKKNGHHIKRHITNDEEKKKEYSFLMLGKEN
RV_3708         MKRFFVLFVIFLVHIWSENVDTFKCNYSKKKKNGHHIKRHITNDEEKKKEYSFLMLGKEN
UGK_408.2       MKRFFVLFVIFLVHIWSENVDTFKCNYSKKKKNGHHIKRHITNDEEKKKEYSFLMLGKEN
309.1           MKRFFVLFVIFLVHIWSENVDTFKCNYSKKKKNGHHIKRHITNDEEKKKEYSFLMLGKEN
UGK_443.2       MKRFFVLFVIFLVHIWSENVDTFKCNYSKKKKNGHHIKRHITNDEEKKKEYSFLMLGKEN
RV_3736         MKRFFVLFVIFLVHIWSENVDTFKCNYSKKKKNGHHIKRHITNDEEKKKEYSFLMLGKEN
PS122_G11       MKRFFVLFVIFLVHIWSENVDTFKCNYSKKKKNGHHIKRHITNDEEKKKEYSFLMLGKEN
RV_3687         MKRFFVLFVIFLVHIWSENVDTFKCNYSKKKKNGHHIKRHITNDEEKKKEYSFLMLGKEN
RV_3610         MKRFFVLFVIFLVHIWSENVDTFKCNYSKKKKNGHHIKRHITNDEEKKKEYSFLMLGKEN
PS250           MKRFFVLFVIFLVHIWSENVDTFKCNYSKKKKNGHHIKRHITNDEEKKKEYSFLMLGKEN
RV_3675         MKRFFVLFVIFLVHIWSENVDTFKCNYSKKKKNGHHIKRHITNDEEKKKEYSFLMLGKEN
RV_3606         MKRFFVLFVIFLVHIWSENVDTFKCNYSKKKKNGHHIKRHITNDEEKKKEYSFLMLGKEN
PS103           MKRFFVLFVIFLVHIWSENVDTFKCNYSKKKKNGHHIKRHITNDEEKKKEYSFLMLGKEN
RV_3741         MKRFFVLFVIFLVHIWSENVDTFKCNYSKKKKNGHHIKRHITNDEEKKKEYSFLMLGKEN
TRIPS_437       MKRFFVLFVIFLVHIWSENVDTFKCNYSKKKKNGHHIKRHITNDEEKKKEYSFLMLGKEN
TRIPS_474       MKRFFVLFVIFLVHIWSENVDTFKCNYSKKKKNGHHIKRHITNDEEKKKEYSFLMLGKEN
RV_3701         MKRFFVLFVIFLVHIWSENVDTFKCNYSKKKKNGHHIKRHITNDEEKKKEYSFLMLGKEN
PS183           MKRFFVLFVIFLVHIWSENVDTFKCNYSKKKKNGHHIKRHITNDEEKKKEYSFLMLGKEN
RV_3731         MKRFFVLFVIFLVHIWSENVDTFKCNYSKKKKNGHHIKRHITNDEEKKKEYSFLMLGKEN
TRIPS_364       MKRFFVLFVIFLVHIWSENVDTFKCNYSKKKKNGHHIKRHITNDEEKKKEYSFLMLGKEN
BM_0009         MKRFFVLFVIFLVHIWSENVDTFKCNYSKKKKNGHHIKRHITNDEEKKKEYSFLMLGKEN
TRIPS_331       MKRFFVLFVIFLVHIWSENVDTFKCNYSKKKKNGHHIKRHITNDEEKKKEYSFLMLGKEN
RV_3766         MKRFFVLFVIFLVHIWSENVDTFKCNYSKKKKNGHHIKRHITNDEEKKKEYSFLMLGKEN
TRIPS_355       MKRFFVLFVIFLVHIWSENVDTFKCNYSKKKKNGHHIKRHITNDEEKKKEYSFLMLGKEN
TRIPS_461       MKRFFVLFVIFLVHIWSENVDTFKCNYSKKKKNGHHIKRHITNDEEKKKEYSFLMLGKEN
TRIPS_501       MKRFFVLFVIFLVHIWSENVDTFKCNYSKKKKNGHHIKRHITNDEEKKKEYSFLMLGKEN
O079-B          MKRFFVLFVIFLVHIWSENVDTFKCNYSKKKKNGHHIKRHITNDEEKKKEYSFLMLGKEN
H209            MKRFFVLFVIFLVHIWSENVDTFKCNYSKKKKNGHHIKRHITNDEEKKKEYSFLMLGKEN
P196J3-C        MKRFFVLFVIFLVHIWSENVDTFKCNYSKKKKNGHHIKRHITNDEEKKKEYSFLMLGKEN
SenT101.09      MKRFFVLFVIFLVHIWSENVDTFKCNYSKKKKNGHHIKRHITNDEEKKKEYSFLMLGKEN
SenT149.09      MKRFFVLFVIFLVHIWSENVDTFKCNYSKKKKNGHHIKRHITNDEEKKKEYSFLMLGKEN
SenT180.08      MKRFFVLFVIFLVHIWSENVDTFKCNYSKKKKNGHHIKRHITNDEEKKKEYSFLMLGKEN
SenT142.09      MKRFFVLFVIFLVHIWSENVDTFKCNYSKKKKNGHHIKRHITNDEEKKKEYSFLMLGKEN
N011-A          MKRFFVLFVIFLVHIWSENVDTFKCNYSKKKKNGHHIKRHITNDEEKKKEYSFLMLGKEN
O306-A          MKRFFVLFVIFLVHIWSENVDTFKCNYSKKKKNGHHIKRHITNDEEKKKEYSFLMLGKEN
P241-D          MKRFFVLFVIFLVHIWSENVDTFKCNYSKKKKNGHHIKRHITNDEEKKKEYSFLMLGKEN
RV_3635         MKRFFVLFVIFLVHIWSENVDTFKCNYSKKKKNGHHIKRHITNDEEKKKEYSFLMLGKEN
P164-C          MKRFFVLFVIFLVHIWSENVDTFKCNYSKKKKNGHHIKRHITNDEEKKKEYSFLMLGKEN
RV_3630         MKRFFVLFVIFLVHIWSENVDTFKCNYSKKKKNGHHIKRHITNDEEKKKEYSFLMLGKEN
RV_3739         MKRFFVLFVIFLVHIWSENVDTFKCNYSKKKKNGHHIKRHITNDEEKKKEYSFLMLGKEN
RV_3730         MKRFFVLFVIFLVHIWSENVDTFKCNYSKKKKNGHHIKRHITNDEEKKKEYSFLMLGKEN
BM_0008         MKRFFVLFVIFLVHIWSENVDTFKCNYSKKKKNGHHIKRHITNDEEKKKEYSFLMLGKEN
TRIPS_759       MKRFFVLFVIFLVHIWSENVDTFKCNYSKKKKNGHHIKRHITNDEEKKKEYSFLMLGKEN
RV_3735         MKRFFVLFVIFLVHIWSENVDTFKCNYSKKKKNGHHIKRHITNDEEKKKEYSFLMLGKEN
TRIPS_440       MKRFFVLFVIFLVHIWSENVDTFKCNYSKKKKNGHHIKRHITNDEEKKKEYSFLMLGKEN
RV_3721         MKRFFVLFVIFLVHIWSENVDTFKCNYSKKKKNGHHIKRHITNDEEKKKEYSFLMLGKEN
P237-C          MKRFFVLFVIFLVHIWSENVDTFKCNYSKKKKNGHHIKRHITNDEEKKKEYSFLMLGKEN
TRIPS_487       MKRFFVLFVIFLVHIWSENVDTFKCNYSKKKKNGHHIKRHITNDEEKKKEYSFLMLGKEN
M113-A          MKRFFVLFVIFLVHIWSENVDTFKCNYSKKKKNGHHIKRHITNDEEKKKEYSFLMLGKEN
RV_3714         MKRFFVLFVIFLVHIWSENVDTFKCNYSKKKKNGHHIKRHITNDEEKKKEYSFLMLGKEN
RV_3717         MKRFFVLFVIFLVHIWSENVDTFKCNYSKKKKNGHHIKRHITNDEEKKKEYSFLMLGKEN
327.1           MKRFFVLFVIFLVHIWSENVDTFKCNYSKKKKNGHHIKRHITNDEEKKKEYSFLMLGKEN
RV_3655         MKRFFVLFVIFLVHIWSENVDTFKCNYSKKKKNGHHIKRHITNDEEKKKEYSFLMLGKEN
                ************************************************************
```

```
                61                                                        120
3D7             EEENKENKENNQNVNKDNNDNNNNEKKNEEQNHNNEKKQEEVINNNNNNVENKKEEENHN
TRIPS_480       EEENKENKENNQNVNKDNNDNNNNEKKNEEQNHNNEKKQEEVINNNNNNVENKKEEENHN
RV_3703         EEENKENKENNQNVNKDNNDNNNNEKKNEEQNHNNEKKQEEVINNNNNNVENKKEEENHN
RV_3637         EEENKENKENNQNVNKDNNDNNNNEKKNEEQNHNNEKKQEEVINNNNNNVENKKEEENHN
RV_3729         EEENKENKENNQNVNKDNNDNNNNEKKNEEQNHNNEKKQEEVINNNNNNVENKKEEENHN
RV_3708         EEENKENKENNQNVNKDNNDNNNNEKKNEEQNHNNEKKQEEVINNNNNNVENKKEEENHN
UGK_408.2       EEENKENKENNQNVNKDNNDNNNNEKKNEEQNHNNEKKQEEVINNNNNNVENKKEEENHN
309.1           EEENKENKENNQNVNKDNNDNNNNEKKNEEQNHNNEKKQEEVINNNNNNVENKKEEENHN
UGK_443.2       EEENKENKENNQNVNKDNNDNNNNEKKNEEQNHNNEKKQEEVINNNNNNVENKKEEENHN
RV_3736         EEENKENKENNQNVNKDNNDNNNNEKKNEEQNHNNEKKQEEVINNNNNNVENKKEEENHN
PS122_G11       EEENKENKENNQNVNKDNNDNNNNEKKNEEQNHNNEKKQEEVINNNNNNVENKKEEENHN
RV_3687         EEENKENKENNQNVNKDNNDNNNNEKKNEEQNHNNEKKQEEVINNNNNNVENKKEEENHN
RV_3610         EEENKENKENNQNVNKDNNDNNNNEKKNEEQNHNNEKKQEEVINNNNNNVENKKEEENHN
PS250           EEENKENKENNQNVNKDNNDNNNNEKKNEEQNHNNEKKQEEVINNNNNNVENKKEEENHN
RV_3675         EEENKENKENNQNVNKDNNDNNNNEKKNEEQNHNNEKKQEEVINNNNNNVENKKEEENHN
RV_3606         EEENKENKENNQNVNKDNNDNNNNEKKNEEQNHNNEKKQEEVINNNNNNVENKKEEENHN
PS103           EEENKENKENNQNVNKDNNDNNNNEKKNEEQNHNNEKKQEEVINNNNNNVENKKEEENHN
RV_3741         EEENKENKENNQNVNKDNNDNNNNEKKNEEQNHNNEKKQEEVINNNNNNVENKKEEENHN
TRIPS_437       EEENKENKENNQNVNKDNNDNNNNEKKNEEQNHNNEKKQEEVINNNNNNVENKKEEENHN
TRIPS_474       EEENKENKENNQNVNKDNNDNNNNEKKNEEQNHNNEKKQEEVINNNNNNVENKKEEENHN
RV_3701         EEENKENKENNQNVNKDNNDNNNNEKKNEEQNHNNEKKQEEVINNNNNNVENKKEEENHN
PS183           EEENKENKENNQNVNKDNNDNNNNEKKNEEQNHNNEKKQEEVINNNNNNVENKKEEENHN
RV_3731         EEENKENKENNQNVNKDNNDNNNNEKKNEEQNHNNEKKQEEVINNNNNNVENKKEEENHN
TRIPS_364       EEENKENKENNQNVNKDNNDNNNNEKKNEEQNHNNEKKQEEVINNNNNNVENKKEEENHN
BM_0009         EEENKENKENNQNVNKDNNDNNNNEKKNEEQNHNNEKKQEEVINNNNNNVENKKEEENHN
TRIPS_331       EEENKENKENNQNVNKDNNDNNNNEKKNEEQNHNNEKKQEEVINNNNNNVENKKEEENHN
RV_3766         EEENKENKENNQNVNKDNNDNNNNEKKNEEQNHNNEKKQEEVINNNNNNVENKKEEENHN
TRIPS_355       EEENKENKENNQNVNKDNNDNNNNEKKNEEQNHNNEKKQEEVINNNNNNVENKKEEENHN
TRIPS_461       EEENKENKENNQNVNKDNNDNNNNEKKNEEQNHNNEKKQEEVINNNNNNVENKKEEENHN
TRIPS_501       EEENKENKENNQNVNKDNNDNNNNEKKNEEQNHNNEKKQEEVINNNNNNVENKKEEENHN
O079-B          EEENKENKENNQNVNKDNNDNNNNEKKNEEQNHNNEKKQEEVINNNNNNVENKKEEENHN
H209            EEENKENKENNQNVNKDNNDNNNNEKKNEEQNHNNEKKQEEVINNNNNNVENKKEEENHN
P196J3-C        EEENKENKENNQNVNKDNNDNNNNEKKNEEQNHNNEKKQEEVINNNNNNVENKKEEENHN
SenT101.09      EEENKENKENNQNVNKDNNDNNNNEKKNEEQNHNNEKKQEEVINNNNNNVENKKEEENHN
SenT149.09      EEENKENKENNQNVNKDNNDNNNNEKKNEEQNHNNEKKQEEVINNNNNNVENKKEEENHN
SenT180.08      EEENKENKENNQNVNKDNNDNNNNEKKNEEQNHNNEKKQEEVINNNNNNVENKKEEENHN
SenT142.09      EEENKENKENNQNVNKDNNDNNNNEKKNEEQNHNNEKKQEEVINNNNNNVENKKEEENHN
N011-A          EEENKENKENNQNVNKDNNDNNNNEKKNEEQNHNNEKKQEEVINNNNNNVENKKEEENHN
O306-A          EEENKENKENNQNVNKDNNDNNNNEKKNEEQNHNNEKKQEEVINNNNNNVENKKEEENHN
P241-D          EEENKENKENNQNVNKDNNDNNNNEKKNEEQNHNNEKKQEEVINNNNNNVENKKEEENHN
RV_3635         EEENKENKENNQNVNKDNNDNNNNEKKNEEQNHNNEKKQEEVINNNNNNVENKKEEENHN
P164-C          EEENKENKENNQNVNKDNNDNNNNEKKNEEQNHNNEKKQEEVINNNNNNVENKKEEENHN
RV_3630         EEENKENKENNQNVNKDNNDNNNNEKKNEEQNHNNEKKQEEVINNNNNNVENKKEEENHN
RV_3739         EEENKENKENNQNVNKDNNDNNNNEKKNEEQNHNNEKKQEEVINNNNNNVENKKEEENHN
RV_3730         EEENKENKENNQNVNKDNNDNNNNEKKNEEQNHNNEKKQEEVINNNNNNVENKKEEENHN
BM_0008         EEENKENKENNQNVNKDNNDNNNNEKKNEEQNHNNEKKQEEVINNNNNNVENKKEEENHN
TRIPS_759       EEENKENKENNQNVNKDNNDNNNNEKKNEEQNHNNEKKQEEVINNNNNNVENKKEEENHN
RV_3735         EEENKENKENNQNVNKDNNDNNNNEKKNEEQNHNNEKKQEEVINNNNNNVENKKEEENHN
TRIPS_440       EEENKENKENNQNVNKDNNDNNNNEKKNEEQNHNNEKKQEEVINNNNNNVENKKEEENHN
RV_3721         EEENKENKENNQNVNKDNNDNNNNEKKNEEQNHNNEKKQEEVINNNNNNVENKKEEENHN
P237-C          EEENKENKENNQNVNKDNNDNNNNEKKNEEQNHNNEKKQEEVINNNNNNVENKKEEENHN
TRIPS_487       EEENKENKENNQNVNKDNNDNNNNEKKNEEQNHNNEKKQEEVINNNNNNVENKKEEENHN
M113-A          EEENKENKENNQNVNKDNNDNNNNEKKNEEQNHNNEKKQEEVINNNNNNVENKKEEENHN
RV_3714         EEENKENKENNQNVNKDNNDNNNNEKKNEEQNHNNEKKQEEVINNNNNNVENKKEEENHN
RV_3717         EEENKENKENNQNVNKDNNDNNNNEKKNEEQNHNNEKKQEEVINNNNNNVENKKEEENHN
327.1           EEENKENKENNQNVNKDNNDNNNNEKKNEEQNHNNEKKQEEVINNNNNNVENKKEEENHN
RV_3655         EEENKENKENNQNVNKDNNDNNNNEKKNEEQNHNNEKKQEEVINNNNNNVENKKEEENHN
                ************************************************************
```

```
                121                                                       180
3D7             NDKKTDEQNHVNEKKQEGENNKHKKDDTIPPEKKINKENLLEYGTHDKEGHFIPSYKTLT
TRIPS_480       NDKKTDEQNHVNEKKQEGENNKHKKDDTIPPEKKINKENLLEYGTHDKEGHFIPSYKTLT
RV_3703         NDKKTDEQNHVNEKKQEGENNKHKKDDTIPPEKKINKENLLEYGTHDKEGHFIPSYKTLT
RV_3637         NDKKTDEQNHVNEKKQEGENNKHKKDDTIPPEKKINKENLLEYGTHDKEGHFIPSYKTLT
RV_3729         NDKKTDEQNHVNEKKQEGENNKHKKDDTIPPEKKINKENLLEYGTHDKEGHFIPSYKTLT
RV_3708         NDKKTDEQNHVNEKKQEGENNKHKKDDTIPPEKKINKENLLEYGTHDKEGHFIPSYKTLT
UGK_408.2       NDKKTDEQNHVNEKKQEGENNKHKKDDTIPPEKKINKENLLEYGTHDKEGHFIPSYKTLT
309.1           NDKKTDEQNHVNEKKQEGENNKHKKDDTIPPEKKINKENLLEYGTHDKEGHFIPSYKTLT
UGK_443.2       NDKKTDEQNHVNEKKQEGENNKHKKDDTIPPEKKINKENLLEYGTHDKEGHFIPSYKTLT
RV_3736         NDKKTDEQNHVNEKKQEGENNKHKKDDTIPPEKKINKENLLEYGTHDKEGHFIPSYKTLT
PS122_G11       NDKKTDEQNHVNEKKQEGENNKHKKDDTIPPEKKINKENLLEYGTHDKEGHFIPSYKTLT
RV_3687         NDKKTDEQNHVNEKKQEGENNKHKKDDTIPPEKKINKENLLEYGTHDKEGHFIPSYKTLT
RV_3610         NDKKTDEQNHVNEKKQEGENNKHKKDDTIPPEKKINKENLLEYGTHDKEGHFIPSYKTLT
PS250           NDKKTDEQNHVNEKKQEGENNKHKKDDTIPPEKKINKENLLEYGTHDKEGHFIPSYKTLT
RV_3675         NDKKTDEQNHVNEKKQEGENNKHKKDDTIPPEKKINKENLLEYGTHDKEGHFIPSYKTLT
RV_3606         NDKKTDEQNHVNEKKQEGENNKHKKDDTIPPEKKINKENLLEYGTHDKEGHFIPSYKTLT
PS103           NDKKTDEQNHVNEKKQEGENNKHKKDDTIPPEKKINKENLLEYGTHDKEGHFIPSYKTLT
RV_3741         NDKKTDEQNHVNEKKQEGENNKHKKDDTIPPEKKINKENLLEYGTHDKEGHFIPSYKTLT
TRIPS_437       NDKKTDEQNHVNEKIQEGENNKHKKDDTIPPEKKINKENLLEYGTHDKEGHFIPSYKTLT
TRIPS_474       NDKKTDEQNHVNEKIQEGENNKHKKDDTIPPEKKINKENLLEYGTHDKEGHFIPSYKTLT
RV_3701         NDKKTDEQNHVNEKIQEGENNKHKKDDTIPPEKKINKENLLEYGTHDKEGHFIPSYKTLT
PS183           NDKKTDEQNHVNEKIQEGENNKHKKDDTIPPEKKINKENLLEYGTHDKEGHFIPSYKTLT
RV_3731         NDKKTDEQNHVNEKKQEGENNKHKKDDTIPPEKKINKENLLEYGTHDKEGHFIPSYKTLT
TRIPS_364       NDKKTDEQNHVNEKKQEGENNKHKKDDTIPPEKKINKENLLEYGTHDKEGHFIPSYKTLT
BM_0009         NDKKTDEQNHVNEKKQEGENNKHKKDDTIPPEKKINKENLLEYGTHDKEGHFIPSYKTLT
TRIPS_331       NDKKTDEQNHVNEKKQEGENNKHKKDDTIPPEKKINKENLLEYGTHDKEGHFIPSYKTLT
RV_3766         NDKKTDEQNHVNEKKQEGENNKHKKDDTIPPEKKINKENLLEYGTHDKEGHFIPSYKTLT
TRIPS_355       NDKKTDEQNHVNEKKQEGENNKHKKDDTIPPEKKINKENLLEYGTHDKEGHFIPSYKTLT
TRIPS_461       NDKKTDEQNHVNEKKQEGENNKHKKDDTIPPEKKINKENLLEYGTHDKEGHFIPSYKTLT
TRIPS_501       NDKKTDEQNHVNEKKQEGENNKHKKDDTIPPEKKINKENLLEYGTHDKEGHFIPSYKTLT
O079-B          NDKKTDEQNHVNEKKQEGENNKHKKDDTIPPEKKINKENLLEYGTHDKEGHFIPSYKTLT
H209            NDKKTDEQNHVNEKKQEGENNKHKKDDTIPPEKKINKENLLEYGTHDKEGHFIPSYKTLT
P196J3-C        NDKKTDEQNHVNEKKQEGENNKHKKDDTIPPEKKINKENLLEYGTHDKEGHFIPSYKTLT
SenT101.09      NDKKTDEQNHVNEKKQEGENNKHKKDDTIPPEKKINKENLLEYGTHDKEGHFIPSYKTLT
SenT149.09      NDKKTDEQNHVNEKKQEGENNKHKKDDTIPPEKKINKENLLEYGTHDKEGHFIPSYKTLT
SenT180.08      NDKKTDEQNHVNEKIQEGENNKHKKDDTIPPEKKINKENLLEYGTHDKEGHFIPSYKTLT
SenT142.09      NDKKTDEQNHVNEKKQEGENNKHKKDDTIPPEKKINKENLLEYGTHDKEGHFIPSYKTLT
N011-A          NDKKTDEQNHVNEKKQEGENNKHKKDDTIPPEKKINKENLLEYGTHDKEGHFIPSYKTLT
O306-A          NDKKTDEQNHVNEKKQEGENNKHKKDDTIPPEKKINKENLLEYGTHDKEGHFIPSYKTLT
P241-D          NDKKTDEQNHVNEKKQEGENNKHKKDDTIPPEKKINKENLLEYGTHDKEGHFIPSYKTLT
RV_3635         NDKKTDEQNHVNEKKQEGENNKHKKDDTIPPEKKINKENLLEYGTHDKEGHFIPSYKTLT
P164-C          NDKKTDEQNHVNEKKQEGENNKHKKDDTIPPEKKINKENLLEYGTHDKEGHFIPSYKTLT
RV_3630         NDKKTDEQNHVNEKIQEGENNKHKKDDTIPPEKKINKENLLEYGTHDKEGHFIPSYKTLT
RV_3739         NDKKTDEQNHVNEKIQEGENNKHKKDDTIPPEKKINKENLLEYGTHDKEGHFIPSYKTLT
RV_3730         NDKKTDEQNHVNEKIQEGENNKHKKDDTIPPEKKINKENLLEYGTHDKEGHFIPSYKTLT
BM_0008         NDKKTDEQNHVNEKIQEGENNKHKKDDTIPPEKKINKENLLEYGTHDKEGHFIPSYKTLT
TRIPS_759       NDKKTDEQNHVNEKIQEGENNKHKKDDTIPPEKKINKENLLEYGTHDKEGHFIPSYKTLT
RV_3735         NDKKTDEQNHVNEKIQEGENNKHKKDDTIPPEKKINKENLLEYGTHDKEGHFIPSYKTLT
TRIPS_440       NDKKTDEQNHVNEKIQEGENNKHKKDDTIPPEKKINKENLLEYGTHDKEGHFIPSYKTLT
RV_3721         NDKKTDEQNHVNEKKQEGENNKHKKDDTIPPEKKINKENLLEYGTHDKEGHFIPSYKTLT
P237-C          NDKKTDEQNHVNEKKQEGENNKHKKDDTIPPEKKINKENLLEYGTHDKEGHFIPSYKTLT
TRIPS_487       NDKKTDEQNHVNEKIQEGENNKHKKDDTIPPEKKINKENLLEYGTHDKEGHFIPSYKTLT
M113-A          NDKKTDEQNHVNEKKQEGENNKHKKDDTIPPEKKINKENLLEYGTHDKEGHFIPSYKTLT
RV_3714         NDKKTDEQNHVNEKIQEGENNKHKKDDTIPPEKKINKENLLEYGTHDKEGHFIPSYKTLT
RV_3717         NDKKTDEQNHVNEKKQEGENNKHKKDDTIPPEKKINKENLLEYGTHDKEGHFIPSYKTLT
327.1           NDKKTDEQNHVNEKKQEGENNKHKKDDTIPPEKKINKENLLEYGTHDKEGHFIPSYKTLT
RV_3655         NDKKTDEQNHVNEKKQEGENNKHKKDDTIPPEKKINKENLLEYGTHDKEGHFIPSYKTLT
                ************** *********************************************
```

```
                181                                                         240
3D7             DEILSTNNAMEKASSFLKIACSHVMKLIEFIPESKLSSQYIKVDNKNIYLKDIAVECQNI
TRIPS_480       DEILSTNNAMEKASSFLKIACSHVMKLIEFIPESKLSSQYIKVDNKNIYLKDIAVECQNI
RV_3703         DEILSTNNAMEKASSFLKISCSHVMKLIEFIPESKLSSQYIKVDNKNIYLKDIAVECQNI
RV_3637         DEILSTNNAMEKASSFLKISCSHVMKLIEFIPESKLSSQYIKVDNKNIYLKDIAVECQNI
RV_3729         DEILSTNNAMEKASSFLKIACSHVMKLIEFIPESKLSSQYIKVDNKNIYLKDIAVECQNI
RV_3708         DEILSTNNAMEKASSFLKISCSHVMKLIEFIPESKLSSQYIKVDNKNIYLKDIAVECQNI
UGK_408.2       DEILSTNNAMEKASSFLKISCSHVMKLIEFIPESKLSSQYIKVDNKNIYLKDIAVECQNI
309.1           DEILSTNNAMEKASSFLKISCSHVMKLIEFIPESKLSSQYIKVDNKNIYLKDIAVECQNI
UGK_443.2       DEILSTNNAMEKASSFLKISCSHVMKLIEFIPESKLSSQYIKVDNKNIYLKDIAVECQNI
RV_3736         DEILSTNNAMEKASSFLKISCSHVMKLIEFIPESKLSSQYIKVDNKNIYLKDIAVECQNI
PS122_G11       DEILSTNNAMEKASSFLKISCSHVMKLIEFIPESKLSSQYIKVDNKNIYLKDIAVECQNI
RV_3687         DEILSTNNAMEKASSFLKISCSHVMKLIEFIPESKLSSQYIKVDNKNIYLKDIAVECQNI
RV_3610         DEILSTNNAMEKASSFLKISCSHVMKLIEFIPESKLSSQYIKVDNKNIYLKDIAVECQNI
PS250           DEILSTNNAMEKASSFLKISCSHVMKLIEFIPESKLSSQYIKVDNKNIYLKDIAVECQNI
RV_3675         DEILSTNNAMEKASSFLKISCSHVMKLIEFIPESKLSSQYIKVDNKNIYLKDIAVECQNI
RV_3606         DEILSTNNAMEKASSFLKISCSHVMKLIEFIPESKLSSQYIKVDNKNIYLKDIAVECQNI
PS103           DEILSTNNAMEKASSFLKISCSHVMKLIEFIPESKLSSQYIKVDNKNIYLKDIAVECQNI
RV_3741         DEILSTNNAMEKASSFLKIACSHVMKLIEFIPESKLSSQYIKVDNKNIYLKDIAVECQNI
TRIPS_437       DEILSTNNAMEKASSFLKISCSHVMKLIEFIPESKLSSQYIKVDNKNIYLKDIAVECQNI
TRIPS_474       DEILSTNNAMEKASSFLKIACSHVMKLIEFIPESKLSSQYIKVDNKNIYLKDIAVECQNI
RV_3701         DEILSTNNAMEKASSFLKIACSHVMKLIEFIPESKLSSQYIKVDNKNIYLKDIAVECQNI
PS183           DEILSTNNAMEKASSFLKIACSHVMKLIEFIPESKLSSQYIKVDNKNIYLKDIAVECQNI
RV_3731         DEILSTNNAMEKASSFLKIACSHVMKLIEFIPESKLSSQYIKVDNKNIYLKDIAVECQNI
TRIPS_364       DEILSTNNAMEKASSFLKIACSHVMKLIEFIPESKLSSQYIKVDNKNIYLKDIAVECQNI
BM_0009         DEILSTNNAMEKASSFLKIACSHVMKLIEFIPESKLSSQYIKVDNKNIYLKDIAVECQNI
TRIPS_331       DEILSTNNAMEKASSFLKIACSHVMKLIEFIPESKLSSQYIKVDNKNIYLKDIAVECQNI
RV_3766         DEILSTNNAMEKASSFLKIACSHVMKLIEFIPESKLSSQYIKVDNKNIYLKDIAVECQNI
TRIPS_355       DEILSTNNAMEKASSFLKIACSHVMKLIEFIPESKLSSQYIKVDNKNIYLKDIAVECQNI
TRIPS_461       DEILSTNNAMEKASSFLKIACSHVMKLIEFIPESKLSSQYIKVDNKNIYLKDIAVECQNI
TRIPS_501       DEILSTNNAMEKASSFLKIACSHVMKLIEFIPESKLSSQYIKVDNKNIYLKDIAVECQNI
O079-B          DEILSTNNAMEKASSFLKIACSHVMKLIEFIPESKLSSQYIKVDNKNIYLKDIAVECQNI
H209            DEILSTNNAMEKASSFLKIACSHVMKLIEFIPESKLSSQYIKVDNKNIYLKDIAVECQNI
P196J3-C        DEILSTNNAMEKASSFLKIACSHVMKLIEFIPESKLSSQYIKVDNKNIYLKDIAVECQNI
SenT101.09      DEILSTNNAMEKASSFLKISCSHVMKLIEFIPESKLSSQYIKVDNKNIYLKDIAVECQNI
SenT149.09      DEILSTNNAMEKASSFLKIACSHVMKLIEFIPESKLSSQYIKVDNKNIYLKDIAVECQNI
SenT180.08      DEILSTNNAMEKASSFLKIACSHVMKLIEFIPESKLSSQYIKVDNKNIYLKDIAVECQNI
SenT142.09      DEILSTNNAMEKASSFLKIACSHVMKLIEFIPESKLSSQYIKVDNKNIYLKDIAVECQNI
N011-A          DEILSTNNAMEKASSFLKIACSHVMKLIEFIPESKLSSQYIKVDNKNIYLKDIAVECQNI
O306-A          DEILSTNNAMEKASSFLKIACSHVMKLIEFIPESKLSSQYIKVDNKNIYLKDIAVECQNI
P241-D          DEILSTNNAMEKASSFLKIACSHVMKLIEFIPESKLSSQYIKVDNKNIYLKDIAVECQNI
RV_3635         DEILSTNNAMEKASSFLKISCSHVMKLIEFIPESKLSSQYIKVDNKNIYLKDIAVECQNI
P164-C          DEILSTNNAMEKASSFLKIACSHVMKLIEFIPESKLSSQYIKVDNKNIYLKDIAVECQNI
RV_3630         DEILSTNNAMEKASSFLKIACSHVMKLIEFIPESKLSSQYIKVDNKNIYLKDIAVECQNI
RV_3739         DEILSTNNAMEKASSFLKIACSHVMKLIEFIPESKLSSQYIKVDNKNIYLKDIAVECQNI
RV_3730         DEILSTNNAMEKASSFLKIACSHVMKLIEFIPESKLSSQYIKVDNKNIYLKDIAVECQNI
BM_0008         DEILSTNNAMEKASSFLKIACSHVMKLIEFIPESKLSSQYIKVDNKNIYLKDIAVECQNI
TRIPS_759       DEILSTNNAMEKASSFLKIACSHVMKLIEFIPESKLSSQYIKVDNKNIYLKDIAVECQNI
RV_3735         DEILSTNNAMEKASSFLKIACSHVMKLIEFIPESKLSSQYIKVDNKNIYLKDIAVECQNI
TRIPS_440       DEILSTNNAMEKASSFLKIACSHVMKLIEFIPESKLSSQYIKVDNKNIYLKDIAVECQNI
RV_3721         DEILSTNNAMEKASSFLKIACSHVMKLIEFIPESKLSSQYIKVDNKNIYLKDIAVECQNI
P237-C          DEILSTNNAMEKASSFLKIACSHVMKLIEFIPESKLSSQYIKVDNKNIYLKDIAVECQNI
TRIPS_487       DEILSTNNAMEKASSFLKIACSHVMKLIEFIPESKLSSQYIKVDNKNIYLKDIAVECQNI
M113-A          DEILSTNNAMEKASSFLKIACSHVMKLIEFIPESKLSSQYIKVDNKNIYLKDIAVECQNI
RV_3714         DEILSTNNAMEKASSFLKIACSHVMKLIEFIPESKLSSQYIKVDNKNIYLKDIAVECQNI
RV_3717         DEILSTNNAMEKASSFLKISCSHVMKLIEFIPESKLSSQYIKVDNKNIYLKDIAVECQNI
327.1           DEILSTNNAMEKASSFLKISCSHVMKLIEFIPESKLSSQYIKVDNKNIYLKDIAVECQNI
RV_3655         DEILSTNNAMEKASSFLKISCSHVMKLIEFIPESKLSSQYIKVDNKNIYLKDIAVECQNI
                ******************* ****************************************
```

```
               241                       267
3D7            YFNLEKFSMTLIVFNSKINKFIYSQEK
TRIPS_480      YFNLEKFSMTLIVFNSKINKFIYSQEK
RV_3703        YFNLEKFSMTLIVFNSKINKFIYSQEK
RV_3637        YFNLEKFSMTLIVFNSKINKFIYSQEK
RV_3729        YFNLEKFSMTLIVFNSKINKFIYSQEK
RV_3708        YFNLEKFSMTLIVFNSKINKFIYSQEK
UGK_408.2      YFNLEKFSMTLIVFNSKINKFIYSQEK
309.1          YFNLEKFSMTLIVFNSKINKFIYSQEK
UGK_443.2      YFNLEKFSMTLIVFNSKINKFIYSQEK
RV_3736        YFNLEKFSMTLIVFNSKINKFIYSQEK
PS122_G11      YFNLEKFSMTLIVFNSKINKFIYSQEK
RV_3687        YFNLEKFSMTLIVFNSKINKFIYSQEK
RV_3610        YFNLEKFSMTLIVFNSKINKFIYSQEK
PS250          YFNLEKFSMTLIVFNSKINKFIYSQEK
RV_3675        YFNLEKFSMTLIVFNSKINKFIYSQEK
RV_3606        YFNLEKFSMTLIVFNSKINKFIYSQEK
PS103          YFNLEKFSMTLIVFNSKINKFIYSQEK
RV_3741        YFNLEKFSMTLIVFNSKINKFIYSQEK
TRIPS_437      YFNLEKFSMTLIVFNSKINKFIYSQEK
TRIPS_474      YFNLEKFSMTLIVFNSKINKFIYSQEK
RV_3701        YFNLEKFSMTLIVFNSKINKFIYSQEK
PS183          YFNLEKFSMTLIVFNSKINKFIYSQEK
RV_3731        YFNLEKFSMTLIVFNSKINKFIYSQEK
TRIPS_364      YFNLEKFSMTLIVFNSKINKFIYSQEK
BM_0009        YFNLEKFSMTLIVFNSKINKFIYSQEK
TRIPS_331      YFNLEKFSMTLIVFNSKINKFIYSQEK
RV_3766        YFNLEKFSMTLIVFNSKINKFIYSQEK
TRIPS_355      YFNLEKFSMTLIVFNSKINKFIYSQEK
TRIPS_461      YFNLEKFSMTLIVFNSKINKFIYSQEK
TRIPS_501      YFNLEKFSMTLIVFNSKINKFIYSQEK
O079-B         YFNLEKFSMTLIVFNSKINKFIYSQEK
H209           YFNLEKFSMTLIVFNSKINKFIYSQEK
P196J3-C       YFNLEKFSMTLIVFNSKINKFIYSQEK
SenT101.09     YFNLEKFSMTLIVFNSKINKFIYSQEK
SenT149.09     YFNLEKFSMTLIVFNSKINKFIYSQEK
SenT180.08     YFNLEKFSMTLIVFNSKINKFIYSQEK
SenT142.09     YFNLEKFSMTLIVFNSKINKFIYSQEK
N011-A         YFNLEKFSMTLIVFNSKINKFIYSQEK
O306-A         YFNLEKFSMTLIVFNSKINKFIYSQEK
P241-D         YFNLEKFSMTLIVFNSKINKFIYSQEK
RV_3635        YFNLEKFSMTLIVFNSKINKFIYSQEK
P164-C         YFNLEKFSMTLIVFNSKINKFIYSQEK
RV_3630        YFNLEKFSMTLIVFNSKINKFIYSQEK
RV_3739        YFNLEKFSMTLIVFNSKINKFIYSQEK
RV_3730        YFNLEKFSMTLIVFNSKINKFIYSQEK
BM_0008        YFNLEKFSMTLIVFNSKINKFIYSQEK
TRIPS_759      YFNLEKFSMTLIVFNSKINKFIYSQEK
RV_3735        YFNLEKFSMTLIVFNSKINKFIYSQEK
TRIPS_440      YFNLEKFSMTLIVFNSKINKFIYSQEK
RV_3721        YFNLEKFSMTLIVFNSKINKFIYSQEK
P237-C         YFNLEKFSMTLIVFNSKINKFIYSQEK
TRIPS_487      YFNLEKFSMTLIVFNSKINKFIYSQEK
M113-A         YFNLEKFSMTLIVFNSKINKFIYSQEK
RV_3714        YFNLEKFSMTLIVFNSKINKFIYSQEK
RV_3717        YFNLEKFSMTLIVFNSKINKFIYSQEK
327.1          YFNLEKFSMTLIVFNSKINKFIYSQEK
RV_3655        YFNLEKFSMTLIVFNSKINKFIYSQEK
               ***************************
```

Supplementary Figure S3: Measurement of the antibody responses (end point titers) against rPfRA. Immunogenicity of rPfRA in (A) mice and (B) rabbit was analyzed by ELISA. Sera were serially diluted and assessed for end point titers. Pre-immune sera were taken as controls. High titer antibodies (end point observed at dilution of 1:320, 000 in mice and 1:640, 000 in rabbit) against the recombinant PfRA protein were detected. The error bars represent the standard error of the mean.

A

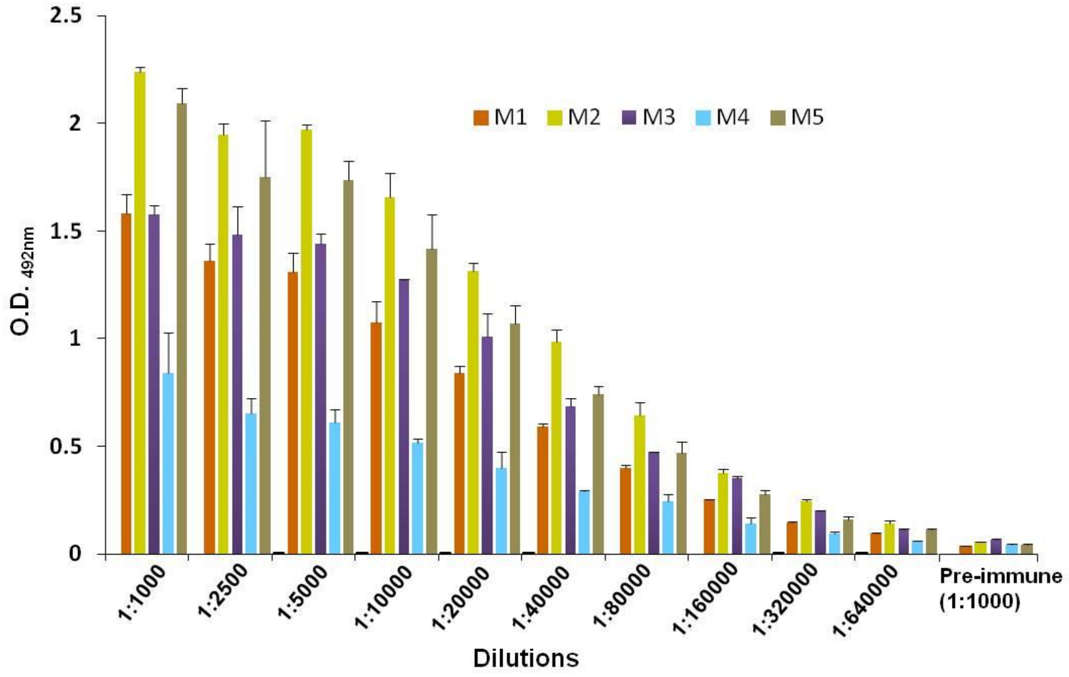

B

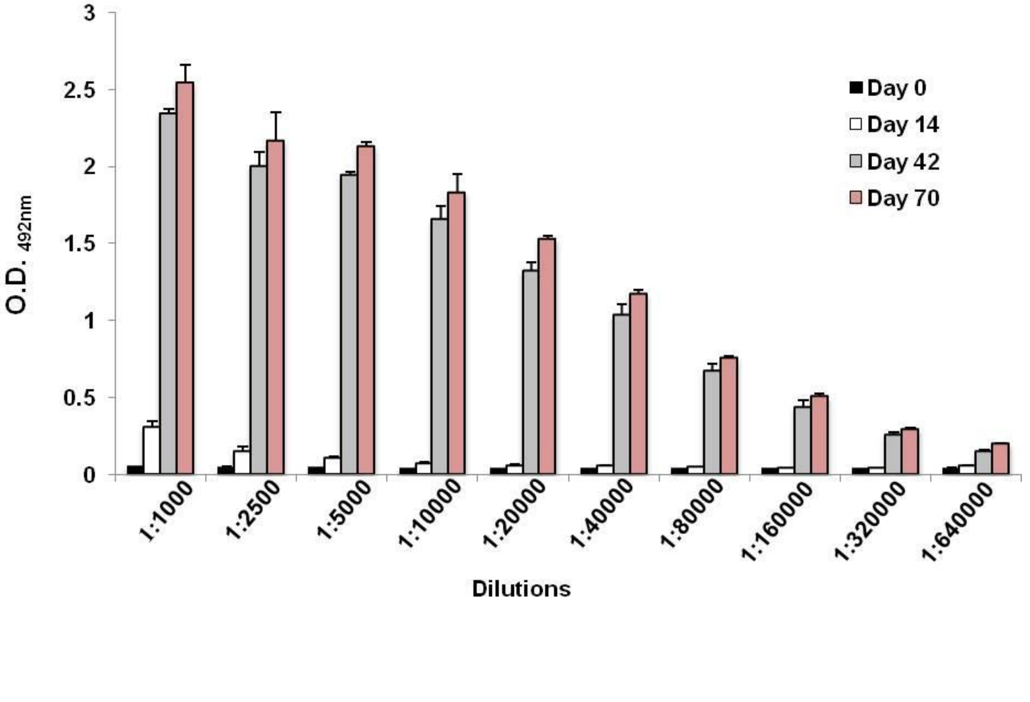

Supplementary Figure S4: Immunoblot analysis of rPfRA with immune sera raised in mice and rabbit. I: immune sera; PI: pre-immune sera.

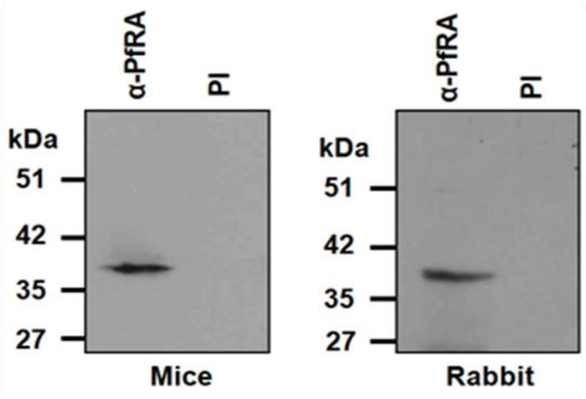

Supplementary Figure S5: Binding of (A) native PfRA and (B) rPfRA to untreated (U), neuraminidase-treated (N), trypsin-treated (T) and chymotrypsin-treated (C) human erythrocytes was detected by flow cytometry using anti-PfRA rabbit sera. Pre-immune rabbit sera (PI) was used as a control.

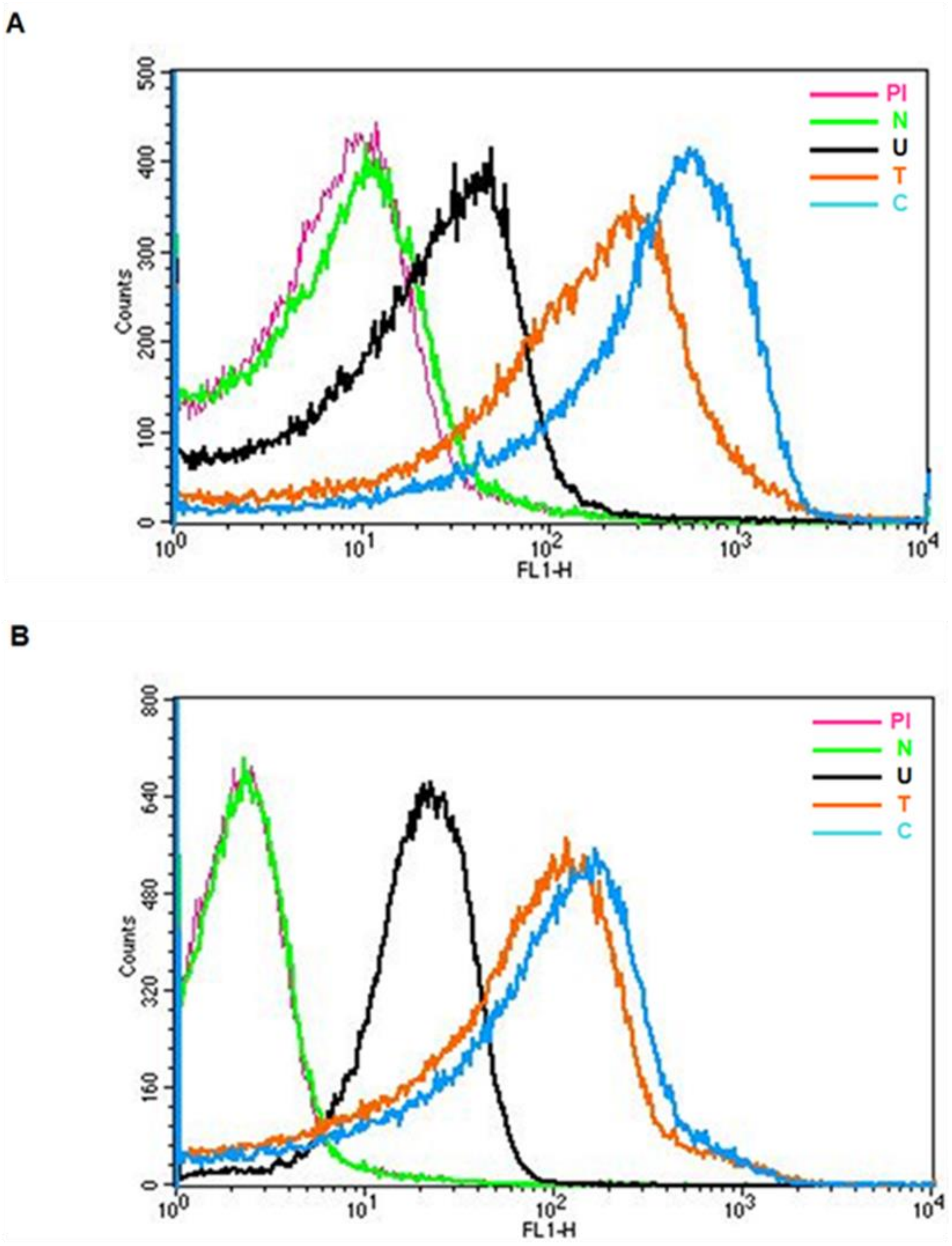

Supplementary Figure S6: Invasion phenotype of three freshly cultured *P. falciparum* field isolates from Manhica, Mozambique in neuraminidase-treated erythrocytes.

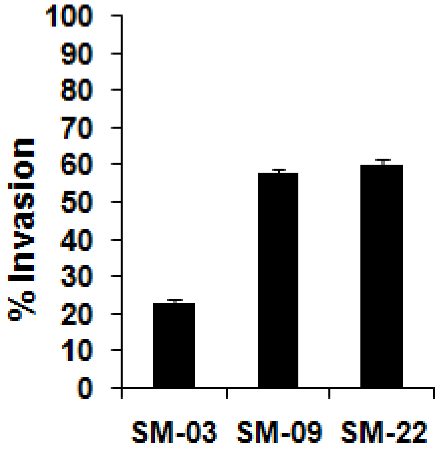

Supplementary Table S1: Geographical origin of *P. falciparum* laboratory clones (www.plasmodb.org).

No.	***P. falciparum* laboratory clone**	**Geographical location**
1.	3D7	Africa
2.	Dd2-1	Laos, Indochina
3.	T9_94	Thailand
4.	Dd2-2	Laos, Indochina
5.	7G8	Brazil
6.	IT	Brazil
7.	707A	Unknown
8.	HB3	Honduras
9.	CS2	Brazil

Supplementary Table S2: Geographical origin of *P. falciparum* field isolates (www.plasmodb.org).

No.	*P. falciparum* field isolate	Geographical location
1.	TRIPS_480	Gambia
2.	RV_3703	Gambia
3.	RV_3637	Gambia
4.	RV_3729	Gambia
5.	RV_3708	Gambia
6.	UGK_408.2	Uganda, Kampala
7.	309.1	Mali
8.	UGK_443.2	Uganda, Kampala
9.	RV_3736	Gambia
10.	PS122_G11	Mali, Bandiagara
11.	RV_3687	Gambia
12.	RV_3610	Gambia
13.	PS250	Mali, Bandiagara
14.	RV_3675	Gambia
15.	RV_3606	Gambia
16.	PS103	Mali, Bandiagara
17.	RV_3741	Gambia
18.	TRIPS_437	Gambia
19.	TRIPS_474	Gambia
20.	RV_3701	Gambia
21.	PS183	Mali, Bandiagara
22.	RV_3731	Gambia
23.	TRIPS_364	Gambia
24.	BM_0009	Gambia
25.	TRIPS_331	Gambia
26.	RV_3766	Gambia
27.	TRIPS_355	Gambia
28.	TRIPS_461	Gambia
29.	TRIPS_501	Gambia
30.	O079-B	French Guiana, Cayenne
31.	H209	French Guiana
32.	P196J3-C	Saint-Laurent du Maroni
33.	SenT101.09	Senegal,Thies
34.	SenT149.09	Senegal,Thies
35.	SenT180.08	Senegal,Thies
36.	SenT142.09	Senegal,Thies
37.	N011-A	French Guiana, Saul
38.	O306-A	French Guiana, Cayenne
40.	RV_3635	Gambia
41.	P164-C	French Guiana, Cayenne

No.	*P. falciparum* field isolate	Geographical location
42.	RV_3630	Gambia
43.	RV_3739	Gambia
44.	RV_3730	Gambia
45.	BM_0008	Gambia
46.	TRIPS_759	Gambia
47.	RV_3735	Gambia
49.	TRIPS_440	Gambia
50.	RV_3721	Gambia
51.	P237-C	French Guiana, Cayenne
52.	TRIPS_487	Gambia
53.	M113-A	Saint Georges de l"Oyapock
54.	RV_3714	Gambia
55.	RV_3717	Gambia
56.	327.1	Mali
57.	RV_3655	Gambia

Supplementary Table S3: List of Unique Peptides generated from the Mass Spectrometric analysis (LC-MS) of the trypsin digested recombinant PfRA protein.

Accession	Description	Score	Coverage
Q8IJS3	Conserved Plasmodium protein OS=Plasmodium falciparum (isolate 3D7) GN=PF10_0119 PE=4 SV=1 - [Q8IJS3_PLAF7]	1226.62	79.03
	A2	Sequence of Unique Peptides	# PSMs
	High	INKENLLEYGTHDKEGHFIPSYK	50
	High	ENEEENKENKENNQNVNK	4
	High	ENLLEYGTHDKEGHFIPSYK	31
	High	KQEEVINNNNNNVENKK	5
	High	EYSFLMLGKENEEENKENK	8
	High	EYSFLmLGKENEEENKENK	16
	High	IAcSHVmKLIEFIPESK	2
	High	LIEFIPESKLSSQYIK	5
	High	KINKENLLEYGTHDK	11
	High	KQEEVINNNNNNVENK	6
	High	EYSFLMLGKENEEENK	3
	High	QEEVINNNNNNVENK	2
	High	KDDTIPPEKK	18
	High	INKENLLEYGTHDK	12
	High	DIAVEcQNIYFNLEK	26
	High	TLTDEILSTNNAMEK	25
	High	EYSFLmLGKENEEENK	11
	High	KNEEQNHNNEK	18
	High	KTDEQNHVNEK	5
	High	TDEQNHVNEK	12
	High	TLTDEILSTNNAmEK	60
	High	VDNKNIYLK	3
	High	KKEYSFLmLGK	8
	High	ENLLEYGTHDK	16
	High	KTDEQNHVNEKK	1
	High	HITNDEEKKK	14

Supplementary Table S4: Mass spectrometric identification of native PfRA from immunoprecipitation elutes using specific antibodies.

Accession	Description	Score	Coverage	Unique Peptides
PF3D7_ 1012200	Organism = Plasmodium_falciparum_3D7| product = conserved Plasmodium protein, unknown function | location = Pf3D7_10_v3:470979 - 471933(+) | length=267 | sequence_SO = chromosome | SO=protein_coding	44.32	22.85	TLTDEILSTnNAMEK
				TLTDEILSTNNAMEK
				DIAVECQNIYFNLEK
				TLTDEILSTNNAmEK
				DIAVEcQNIYFNLEK
				FSMTLIVFNSK
				LIEFIPESK
				HKKDDTIPPEK
				FSMTLIVFnSK

PfRA protein sequence: Unique peptides detected by LC-MS highlighted in green

Signal Sequence (1-22)

MKRFFVLFVIFLVHIWSENVDTFKCNYSKKKKNGHHIKRHITNDEEKKKEYSFLMLGKE

NEEENKENKENNQNVNKDNNDNNNNEKKNEEQNHNNEKKQEEVINNNNNNVENKKE

EENHNNDKKDEQNHVNEKKQEGENNKHKKDDTIPPEKKINKENLLEYGTHDKEGHFIP

SYKTLTDEILSTNNAMEKASSFLKIACSHVMKLIEFIPESKLSSQYIKVDNKNIYLKDIAVE

CQNIYFNLEKFSMTLIVFNSKINKFIYSQEK
